# JuRa: The Juventas Radar on Hera Mission to Probe Internal Structure of Didymos and Dimorphos Asteroids

**DOI:** 10.1007/s11214-026-01324-4

**Published:** 2026-09-21

**Authors:** Alain Herique, Dirk Plettemeier, Yves Rogez, Yann Berquin, Valérie Ciarletti, Wenzhe Fa, Christelle Eyraud, Mark Haynes, Essam Heggy, Amine Kechouindi, Takao Kobayashi, Wlodek Kofman, Matin Laabs, Dominik Nolbert, Elena Pettinelli, Sampsa Pursiainen, Sylvain Rochat, Christian Schmidt, Anne Virkki, Filip Záplata, Sonia Zine, Paul Abell, Ian Carnelli, Hannah Goldberg, Simon Green, Michael Küppers, Monica Lazzarin, Patrick Michel, Franco Perez Lissi, Seiji Sugita, Stephan Ulamec

**Affiliations:** 1 CNRS, CNES, IPAG, Univ. Grenoble Alpes, 38000 Grenoble, France; 2 Technische Universität Dresden, 01069 Dresden, Germany; 3 LATMOS/IPSL, UVSQ Université Paris-Saclay, Sorbonne Université, CNRS, Guyancourt, France; 4 School of Earth and Space Sciences, Peking University, China, 100871 Beijing, China; 5 Centrale Med, Institut Fresnel, Aix Marseille Univ, CNRS, Marseille, France; 6 Jet Propulsion Laboratory, California Institute of Technology, Pasadena, CA 91109 USA; 7 Viterbi School of Engineering, University of Southern California, Los Angeles, CA USA; 8 EmTroniX Sàrl, Building SISA, 5 Rue Bommel, L-4940 Hautcharage, Luxembourg; 9 Korea Institute of Geoscience and Mineral Resources, Yuseong, Daejeon 34132 Korea; 10 Centrum Badan Kosmicznych Polskiej Akademii Nauk (CBK PAN), Bartycka 18A, Warsaw, Poland; 11 Astronika sp. z o.o., Słowikowskiego 81A, 02-793 Warsaw, Poland; 12 Roma Tre University, Rome, Italy; 13 Mathematics Research Center, Computing Sciences, Tampere University, Korkeakoulunkatu 1, 33720 Tampere, Finland; 14 Department of Physics, University of Helsinki, Gustaf Hällströmin katu 2, 00014 Helsingin yliopisto, Finland; 15 FSatCom, Brno, 61200 Czech Republic; 16 Astromaterials Research and Exploration Science Division, NASA Johnson Space Center, Houston, TX 77058 USA; 17 European Space Agency (ESA), European Space Research and Technology Center (ESTEC), Keplerlaan 1, 2201 AZ Noordwijk, The Netherlands; 18 HE Space Operations B.V. for the European Space Agency, Noordwijk, The Netherlands; 19 School of Physical Sciences, The Open University, Walton Hall, Milton Keynes, MK7 6AA UK; 20 European Space Agency (ESA), European Space Astronomy Centre (ESAC), Camino Bajo del Castillo s/n, 28692 Villanueva de la Cañada, Madrid Spain; 21 Department of Physics, Astronomy-Padova University, Vicolo dell’Osservatorio 3, 35122 Padova, Italy; 22 Observatoire de la Côte d’Azur, CNRS, Laboratoire Lagrange, Université Côte d’Azur, CS 34229, 06304 Nice Cedex 4, France; 23 Dept. of Systems Innovation, School of Engineering, University of Tokyo, Hongo 7-3-1, Bunkyo, Tokyo, 113-0033 Japan; 24 Dept. of Earth and Planetary Science, University of Tokyo, 7-3-1 Hongo, Bunkyo, Tokyo, 113-0033 Japan; 25 German Aerospace Center, DLR-MUSC, Linder Hohe, 51147 Cologne, Germany

**Keywords:** Hera, Didymos, Dimorphos, Radar, Asteroid, Internal structure

## Abstract

JuRa, the Juventas Radar, is the sounding radar of the Hera/ESA mission on cruise to the asteroid Didymos and its moon, Dimorphos. In 2027, it will, for the first time, do direct observation of the internal structure of an asteroid for the benefit of both science and planetary defense. The instrument is a 60 MHz monostatic radar with a 20 MHz bandwidth, operating in full linear polarization, onboard Juventas, a CubeSat that will operate from end of January to end of March 2027, in a sun-synchronous orbit with a radius ranging from 3.3 to 2 km. With an expected penetration depth of 100 meters or more, it will uncover the expected rubble pile structures of Didymos and Dimorphos, detect the presence of internal structures, and characterize the size and the level of heterogeneity of the constituent materials. This knowledge will provide a better understanding of the formation and stability conditions of a binary asteroids, and constrain physical models of accretion and evolution. This article describes the concept of radar sounding and tomographic imaging, then presents the Didymos and Dimorphos system and the objectives of JuRa. It then describes the JuRa’s specifications and design.

## Introduction

On October 7, 2024, the Hera/ESA spacecraft was launched from Cape Canaveral onboard a Falcon 9 rocket from the SpaceX company and is now on cruise to the Didymos Asteroid and its small moon Dimorphos (Michel et al. 2022), which was impacted by the DART / NASA spacecraft on September 2022 (Cheng et al. 2023). After a successful flyby of Mars and Deimos on March 12, 2025, Hera will rendezvous Didymos in the fall of 2026 for six months of observation. The spacecraft carries a suite of scientific instruments described in this article collection, including two CubeSats called Juventas and Milani.

One of Hera’s main objectives is to study the internal structure of Didymos and Dimorphos. This is the ambition of JuRa, the Juventas Radar onboard the Juventas CubeSat. Other measurments will contribute to the knowledge of the internal structure, using mainly radio science, the inter-satellite link (ISL) between Hera and its two CubeSats and a gravimeter. Optical observation through the study of dynamical modes or the analysis of surface features will also contribute to this knowledge. It should be kept in mind that all these approaches are complementary: each opens a different window onto the interior of these bodies, allowing access to a few physical quantities within a limited spatial scale range. Understanding the internal structure requires synergies between these different approaches.

### JuRa

**Fig. 1:**
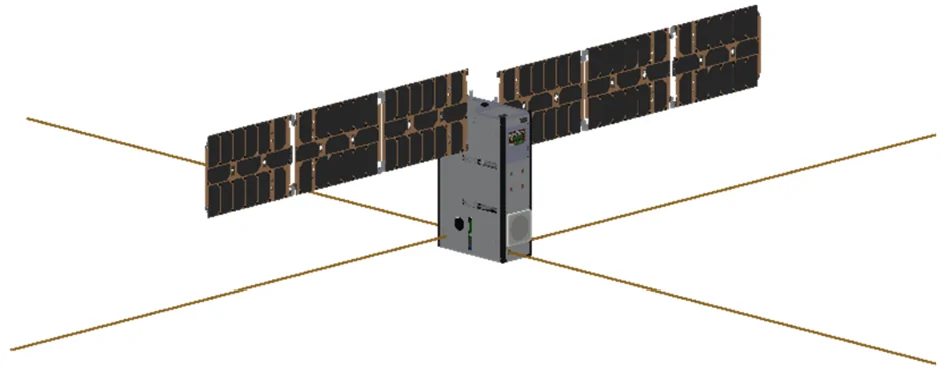
Juventas in flight configuration with all 4 JuRa antenna booms deployed. (courtesy of GomSpace)

Low frequency radar is a mature technique to probe planetary surfaces: starting with the radar sounding of the lunar subsurface with the ALSE experiment onboard Apollo 17 in the seventies (Peeples et al. 1978), it was not until the 2000s that orbital radar was used extensively around the Moon with the LSR instrument on Kaguya-Selene/JAXA (Ono et al. 2009), then around Mars with MARSIS on Mars Express/ESA (Picardi et al. 2005) and SHARAD on MRO/NASA (Seu et al. 2007). In the coming decade the REASON instrument on Europa Clipper/NASA (Blankenship et al. 2024) and RIME on JUICE/ESA (Bruzzone et al. 2015) will reveal Europa’s and Ganymede’s subsurface. Regarding small bodies, the CONSERT instrument on Rosetta has been the only experience to date, probing the interior of the 67P comet nucleus (Kofman et al. 1998, 2015): CONSERT is a very specific radar experiment operating in transmission with a bistatic configuration taking advantage of the high porosity and low contrast of the comet nucleus to perform projection tomography. Several radar experiments have been proposed to probe asteroids, performing diffraction tomography with different resolution and investigation depth (Herique et al. 2018), especially -but not only- in the context of the ESA Cosmic Vision M mission proposals (Oberst et al. 2018; Snodgrass et al. 2018) including the proposed Marco Polo sample return mission (Barucci et al. 2009, 2012; Franchi et al. 2017) and its FANTINA radar package (Herique et al. 2012). These instrument proposals were made possible by Research and Technology studies supported by the French space agency CNES. The radar package considered for the AIDA AIM mission (Michel et al. 2016, 2018) was directly inheriting from FANTINA with a monostatic high frequency radar to probe the shallow subsurface at high resolution (Herique et al. 2019, 2018) and a Low Frequency bistatic Radar carried by a small lander based on the MASCOT lander onboard the Hayabusa 2/JAXA mission to probe the deep interior (Biele et al. 2017; Herique et al. 2019; Lange et al. 2018). JuRa, the monostatic version derived from LFR, was finally considered for the Hera mission (Michel et al. 2022) for implementation on the Juventas CubeSat (Goldberg et al. 2019) . This instrument is a monostatic radar with 20 MHz bandwidth centered around 60 MHz carrier frequency with full polarization capacities (Fig. 1).

### Juventas

Juventas, JuRa carrier, is a 6U CubeSat developed by GOMSpace in a new space approach. 1U is allocated to JuRa electronics in addition to its antenna.

**Fig. 2:**
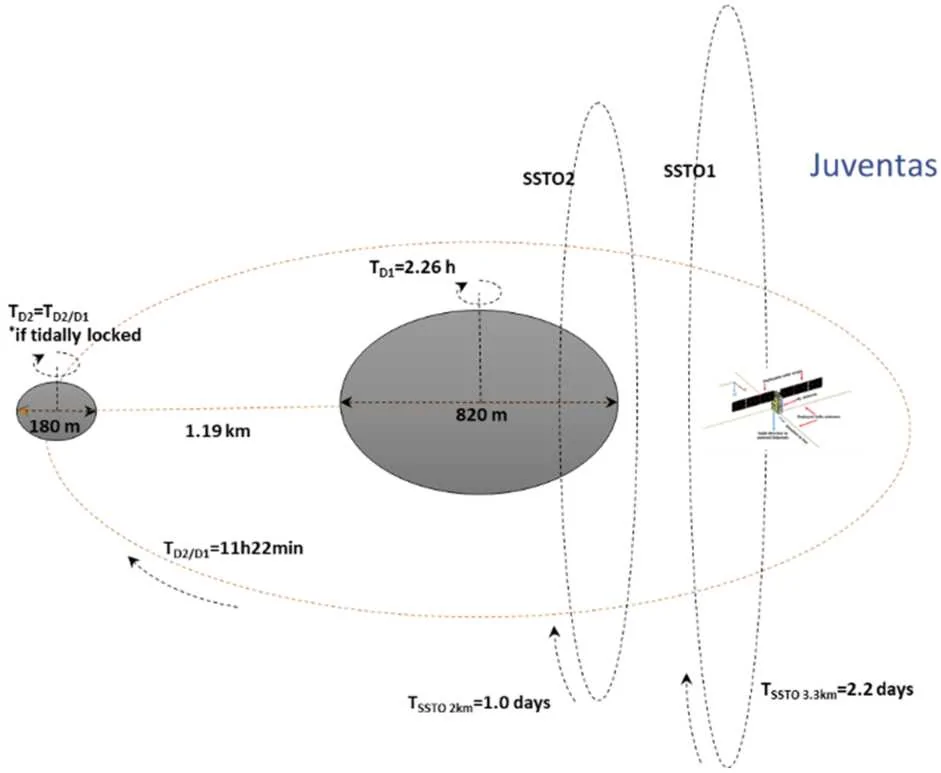
Juventas SSTO orbits phase 1 and 2 (Copyright ©2024 by CNES)

Juventas will be released by Hera in mid-January 2027 to orbit the Didymos binary system (Table 1). After the platform and instrument commissioning and especially once JuRa’s successful antenna deployment followed by a first science acquisition are completed, Juventas will reach a Sun Stabilized Terminator Orbit (SSTO) at the end of January 2027 for several months of science operation at close distance from the asteroid (Fig. 2). It will evolve on a slightly elliptical orbit around the system at a radius of ∼3.3 km for the first month and ∼2 km for the second one. During these two months, JuRa will share operations with the platform as well as the radio-science experiment using the ISL sub-system. At the end of March 2027, Juventas will land on Dimorphos surface for one day of operation dedicated mainly to the gravimeter GRASS. JuRa can operate during descent and possibly after landing if there is still power available after the GRASS operations.

**Table 1:** Juventas mission after-cruise phases in 2027 (start date, duration and distance to Didymos mass center)

PREP	PREparation Phase before release	13 JAN	3 days	10 km
COMP	Release and COMmissioning Phase	16 JAN	3.5 days	10 km
INSP	INSertion Phase	20 JAN	7 days	10 to 3.3 km
SSTO1	Sun Stabilized Terminator Orbit phase 1	27 JAN	30 days	3.3 km radius
TRFP	TRansFer Phase	26 FEB	<1 days	3.3 to 2 km
SSTO2	Sun Stabilized Terminator Orbit phase 2	26 FEB	30 days	2 km radius
LAND	LANDing Phase for surface operations	28 MAR	<1 days	to Dimorphos

A driver to JuRa operation will be the data volume: JuRa telemetry is transmitted from Juventas to the Hera mothercraft using the ISL sub-system. JuRa data volume will be limited by the Hera-to-Earth link. The warranty allocation was 7.8 Gbit for JuRa at the beginning of the project while final allocation would be larger than 20Gbits.

## Asteroid Radar Tomography

### Probing Asteroid Deep Interior

What is JuRa measuring? What is it sensitive to? And what are the retrieved physical quantities? Starting from the basics for those not familiar with radar and wave propagation physics, wave propagation and accordingly any radar measurement are fully defined by the complex permittivity of materials and its spatial variations: the real part of the permittivity $\varepsilon^{\prime }$ defines the wave velocity for material with low dielectric losses while the imaginary part $\varepsilon^{\prime \prime }$, usually represented by the loss tangent tan $\delta = \varepsilon ''$/$\varepsilon^{\prime }$, is related to the dielectric wave losses. JuRa with 20 MHz bandwidth for a 60 MHz carrier frequency (i.e. 5 m wavelength in free space) is measuring the wave reflected and scattered by the surface and the internal heterogeneities of Didymos and Dimorphos.

The propagation regime depends on the heterogeneity’s scale (i.e. size) and the permittivity contrast. For discontinuous material media separated by interfaces such as blocks and voids, heterogeneities smaller than $\sim \lambda $/20 (where λ is the wavelength) are seen as a continuous medium where wave propagation is governed by the geometric optics regime, with an equivalent permittivity depending on the permittivity and shape of the constitutive material (Sihvola 1999): in other words, sand or gravel do not scatter JuRa waves. On the other hand, blocks larger than 20λ are seen as a collection of homogeneous media with geometric optics propagation regime inside blocks and wave refraction, reflection and scattering occurring at their surface: this could be the case if Didymos presents large monolithic sub-aggregates or voids hidden by a regolith blanket. Between theses end-cases, the propagation regime is dominated by scattering: transmitted waves lose their coherence as they propagate inside the bodies and their power is scattered in all the directions. This is typically what is expected from configurations with blocks of several meters separated by metric voids. To complete the story, the power of scattered waves depends on the permittivity contrast between the different constitutive materials: the classical limit at λ/20 – 20λ characterizing the scattering regime depends in practice on the permittivity contrast. Large blocks with voids in-between produce more scattering than the same blocks embedded in sand and gravel. This is one of the physical processes that will constrain the asteroid internal structure.

Elsewhere, inside the same material -rock or sand seen as a continuous medium- smooth variations of the composition and thus of the permittivity also produces scattering. Similarly to above, the scattered power depends on the scale of heterogeneities given by the relative gradient of the permittivity $\frac{\left \vert \overline{\nabla} \varepsilon \right \vert}{\varepsilon} = \frac{1}{l}$ and is negligible when $l \gg \lambda $ which corresponds to the validity domain of the WKB development in geometric optics (Chew 1999).

With JuRa in orbit around the Didymos system, the propagation regime is expected to be dominated by scattering: the generated data will correspond to a map of the returned power per surface or volume unit, quantified by the backscatter coefficient (sigma-null noted $\sigma _{0}$) also called permittivity contrast. The tricky point stems from the lack of knowledge of the internal structure which determines the propagation regime, especially the scattering level. The propagation regime defines the structure of the received signal and the probing depth. It therefore constrains the inversion methods which can be used, the retrieved physical quantities and finally the overall performances of the instrument. Thus, the ability to probe the entirety of Dimorphos by detecting the echo coming from the opposite side mainly depends on the level of volume scattering, which is unknown: a fine material filling the gaps between blocs would limit the level of scattering allowing a direct measurement of the average dielectric permittivity from the absolute propagation delay while metric voids between metrics blocs would limits the resolution and the investigation depth. For this reason, assessing the final performances implicitly requires a model of the internal structure. The preparation of the science analysis, the data processing validation and products definition remain prospective and based on hypothesis. They will be tuned a posteriori and perhaps significantly revisited.

### Geometry of Observations

The instrument final performances and especially the image resolution are largely driven by the diversity of the geometry of acquisition. One radar sounding consists in transmitting a radar wave and receiving the scattered/reflected power as a function of the propagation delay. JuRa antennas provide nearly no directivity, as a result the quasi-totality of Dimorphos and Didymos are observed in a given acquisition, mixing signals arriving with the same propagation time from different asteroid’s features. Only changes of the radar geometry allows the reconstruction of an image as usually done with Synthetic Aperture Radar (SAR) (Curlander and McDonough 1991): a sequence of observation allows to generate 2D image mixing surface and subsurface features in the same pixel while a multipath processing is required to achieve 3D tomography.

The link between geometric diversity and final resolution can be established in the simplest formalism when assuming single scattering propagation regime. The field received by a monostatic radar at position  is then  where  indicates the scatters positions,  the incident field received by the scatters,  the wave vector which is collinear to the propagation direction from  to  and  the contrast function which is equal to  under Born approximation; the factor 2 in the exponential denotes the back and forth propagation in the monostatic configuration (Ishimaru 1999; Kong 2000). In the Fraunhofer approximation (i.e., radar far enough from the asteroid),  with  and . This last expression can be seen as a 3D Fourier transform from the space domain to the  domain. One measurement from one position of the spacecraft gives  the values of the 3D spectrum of the contrast function  for . For a given frequency, f, a monostatic radar can only access  points located on a sphere of radius $2 k = {4 \pi f} / {c_{o}}$ (where $c_{0}$ is the speed of light in vacuum), named the Ewald sphere, while the radar bandwidth gives its thickness to the Ewald sphere and then contributes to improve the final 3D resolution (Haynes et al. 2021; Kofman and Safaeinili 2004). Under this model, the permittivity-contrast map is immediately obtained by the 3D inverse Fourier transform of the measurement.

In practice, the Fraunhofer condition is far from being met when a spacecraft is orbiting a small body at low distance. The processing takes into account this violation as explained hereafter. Single scattering is also a largely questionable hypothesis especially due to refraction at the asteroid surface. For a real asteroid, wave absorption due to both dielectric and scattering losses limits the propagation depth: any position of the spacecraft only probes the facing fraction of the body, especially when observing Didymos, in other words only a fraction of the Ewald sphere / orbit position contributes to the tomography of a given area of the body. Nevertheless, this first order model gives the intuitive path between radar coverage and final 3D resolution, providing an efficient way to select orbits for operation planning. In theory a 3D resolution in the order of half the radar wavelength for radar tomography could be achieved, while in practice the resolution will obviously be limited by the orbit coverage which will be far from fulfilling the Nyquist criteria in space and will not allow coverage of the whole sphere.

**Fig. 3:**
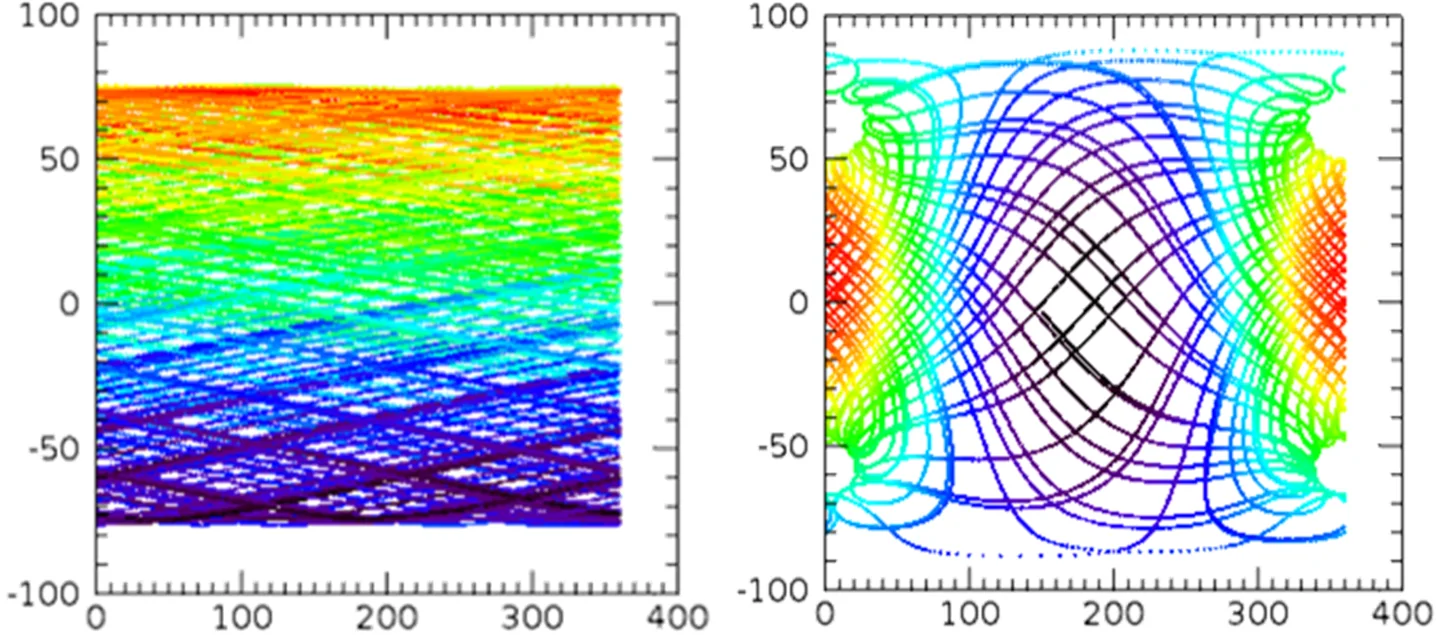
Skymap (lat/long): Juventas trajectory as seen from a point located on the surface of Didymos (left) and Dimorphos (right) for SSTO orbit at 2 km (phase 2), assuming a locked Dimorphos spin. The color represents the altitude

**Fig. 4:**
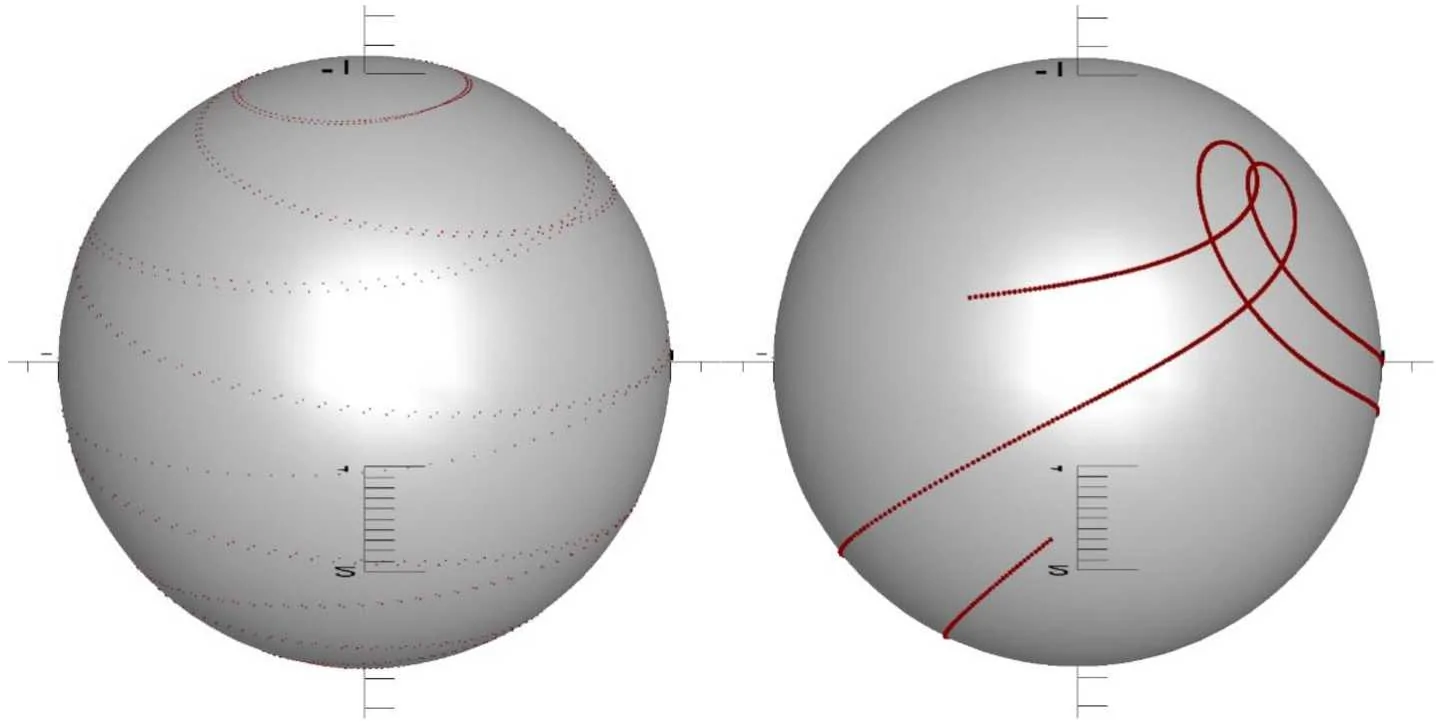
Coverage of the Ewald spheres (k-domain) for an arc of orbit of 24 hours: the different coverage for Didymos (left) and Dimorphos (top, assuming a locked Dimorphos spin) reflects the proper dynamics of each body. (Credit P. Torrente, IPAG)

**Fig. 5:**
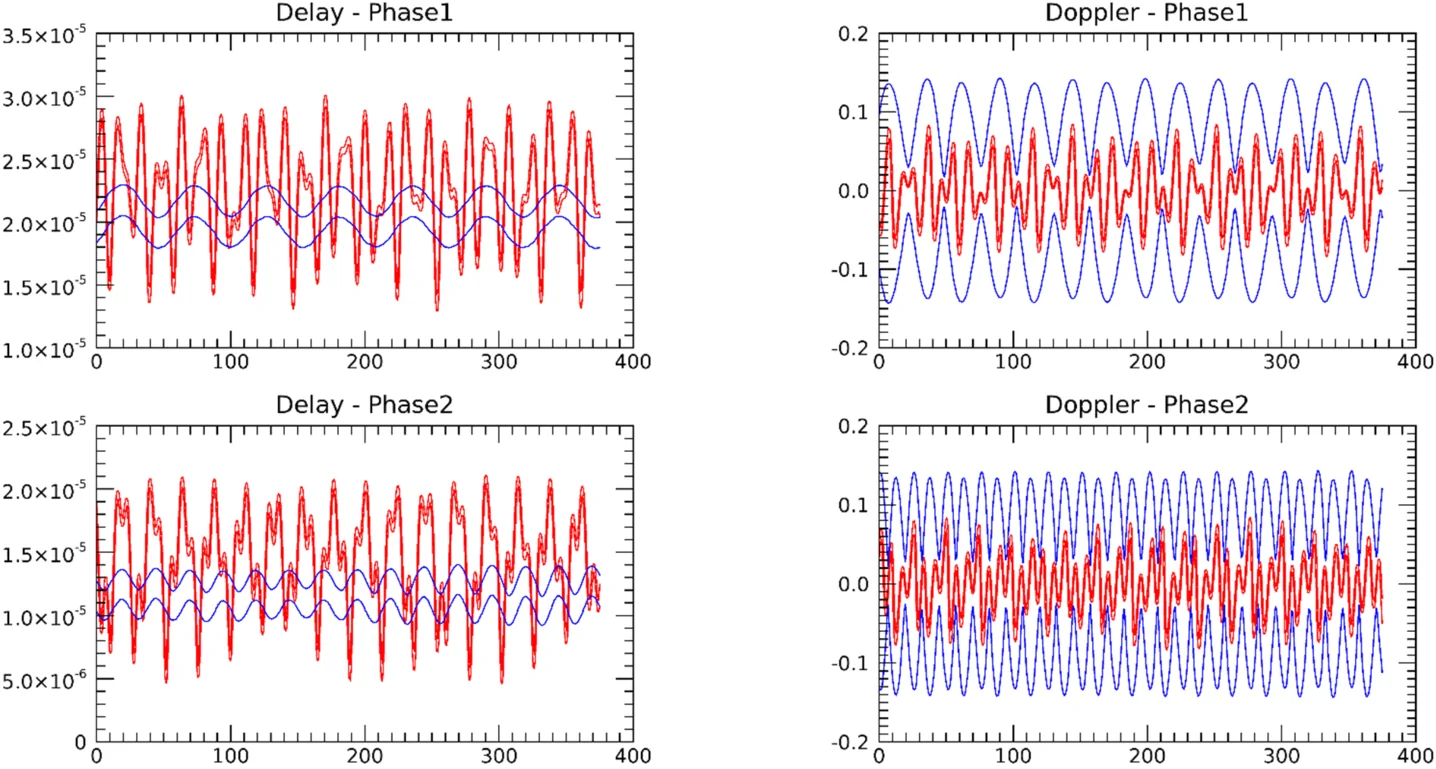
Plot of delay min/max (left in second) and Doppler min / max (right in Hertz), as a function of the number of the 45 minutes acquisition sequence for Dimorphos (red) and Didymos (blue) and for phase 1 (top) and 2 (bottom)

The small-body geometric specificity for radar observation comes also from the rotation of the observed bodies which is higher or at least of the same order of magnitude than the orbital speed of the spacecraft. In our case, the Didymos spin period is 2.26 h, the Dimorphos orbital period is 11 h 22 min with a spin assumed in 1:1 resonance, while the Juventas orbital period is expected to range from 12 h to 18 h for 2 km and 3 km orbit radius respectively. The combination of the two speeds should be considered for any processing done in the body fixed frame, which is different for Didymos and Dimorphos. The spacecraft trajectory is totally different in the two frames (Fig. 3): the Time-Doppler history (Fig. 5) makes the separation of the two bodies possible by processing while the coverage of the Ewald Sphere is then different for Didymos and Dimorphos (Fig. 4) due to different final image resolution. Both sky-maps exhibit cycles and subcycles depending on the ratio spin/orbital periods. For Dimorphos, this regularity is due to the assumed 1:1 resonance which means that a given ground position can only be accessed by one specific configuration of Didymos, Dimorphos and Juventas. As discussed below (Sect. 3.1), Dimorphos could be in excited rotational mode in 2027: this would undoubtedly complicate science observation planning and operations, but would also facilitate uniform radar-coverage of Dimorphos over 2 months due to the avoidance of orbital cycles.

To conclude, it is also worth noting that the considered speeds are of less than a meter per second or a few meters per second, whereas they are about kilometers per second for any radar orbiting a planet.

### Image Formation and SAR Processing

The JuRa processing is just outlined here and will be developed in separate papers. To answer a frequently asked question, JuRa tomography is radically different from the methods developed for CONSERT/Rosetta (Kofman et al. 2015, 2007): JuRa signal will be dominated by scattering corresponding to diffraction tomography as underlined by the Ewald Sphere formalism while 67P comet, which is more homogeneous and corresponds to projection tomography (for CONSERT: Herique et al. 1999; Kofman et al. 2020, for tomography concepts: Chew 1999). The JuRa processing is currently under development. A first family of methods developed for JuRa radar processing is based on synthetic aperture radar processing (Curlander and McDonough 1991): it will serve as the baseline during JuRa science operation to provide the first results in near-real-time and to optimize the next acquisitions. Advanced tomographic methods as presented in the next section are more prospective and will require a posterior validation and tuning.

SAR processing fundamentally consists in a conversion from the signal received in the time domain (propagation delay for different positions of the sensor along the orbit) to a 2D image in the space domain (along track versus across track). The trick is to describe the two-ways propagation delay as a one-way propagation delay at half the wave speed, known as the “exploding reflector model” (Gray et al. 2001): the reflected signal turns into a signal emitted by the scatterers at time t = 0 and propagated from the scatterers to the radar at half the actual wave speed. Accordingly, SAR processing can be seen as a backpropagation (Devaney 2012). For each point of the resulting image, the signals are summed after compensating for the propagation delay to the spacecraft in free space:  where Γ describes the set of measurement.  is proportional to the emitted field and to the backscattering coefficient ($\sigma _{0}$) which characterizes the ability to scatter radar waves and corresponds to the contrast function as define in Sect. 2.1. Outside the Fraunhofer domain, the exponential is a function of  instead of  but we obtain for each position an expression similar to the inverse Fourier transform in the Ewald Sphere formalism. In practice, this equation needs to take into account the calibration and in particular the antenna pattern. For straight line trajectories, SAR synthesis provides resolution in the along-track direction (also referred to as azimuth, Doppler, or slow-time direction), while the RF bandwidth of each radar pulse determines the resolution in the across-track direction (known as range, delay, or fast-time direction). In the small body geometry, orbital arcs are curved mixing range and azimuth resolution in the final image.

Several processing methods to synthetize 2D images have been developed from the end of 1970s in the context of Earth Observation from space and from planes (Tomiyasu 1978). Based on various formalisms, these algorithms find their roots in similar equations and tend to reduce the computation cost through simplification mainly using straight-line geometry of observation and narrow-band property in RF and Doppler domain. Firstly, a straight-line motion of the sensor in the body fixed frame induces a full symmetry of revolution of the solution around the spacecraft trajectory. The processing gives a 2D image easily projected on a plane tangential to the planet surface. Secondly, assuming that the RF bandwidth is narrow compared to the operating frequency (BW/$f_{0}$ ≪ 1) and the antenna beam pattern is narrow (antenna size ≫ wavelength) the target can be observed with a constant group delay over the domain of measurement in incidence/Doppler. This leads to the separability of SAR processing (Curlander and McDonough 1991): the 2D processing can be written as two 1D inverse Fourier transform applied successively in the along-track and in the across-track directions, reducing significantly the algorithm complexity. Numerous methods have been developed to compensate distortions induced by the violation of the separability hypothesis in order to preserve as far as possible the computational efficiency of 2-times 1D processing when RF bandwidths or antenna aperture becomes larger or when trajectory geometry deviates from a straight-line. This includes range migration, processing per-subaperture and autofocus algorithms (Carrara 1995).

Neither of these approximations is valid for JuRa, as BW/$f_{0}$ ∼ 1/3 and the antenna is the size of the wavelength but above all the orbital arcs are curved in the body fixed frame (Fig. 2), mixing range and azimuth resolution in the final image. Therefore, JuRa processing relies on brute-force SAR processing in a 3D world based on previous equations (Sect. 2.2) without any approximation, evaluating the geometry separately for each point of the imaged asteroid (Gassot et al. 2020). This approach allows us to separate the consequences of this non-usual geometry of observation from the effect of any hypothesis violation and to not get lost in the compensation and adaptation of existing algorithms. This full treatment requires significantly more computationally intensive than normal SAR processing, but given the data volume limits due to downlink allocation for a small body mission like Hera, the total computational requirements are manageable.

When processing only one sequence of measurement, the computed image is poorly resolved in the “third” direction (direction orthogonal to the orbit ground track and to the line of sight). Multipass processing is required to resolve the scene. 3D SAR processing inheriting from Earth Observation like 3D backpropagation or compressive sensing was initially considered in the context of High Frequency Radar of the AIDA/AIM project (Gassot et al. 2021): these methods are efficient to focus waves and resolve the image especially when taking into account an average wave speed inside the asteroid.

For the application to JuRa and Dimorphos, Dimorphos’ surface curvature at the scale of the radar field of view involved in the processing (or radar synthetic length) is not at all negligible compared to the wavelength making impossible any planar approximation. The resulting image is unfocused by refraction as the image of a fish in a spherical aquarium. Advanced tomography methods presented hereafter are under development to take into account surface refraction. In parallel, we are considering autofocus techniques (Carrara 1995) to improve orbit restitution and in particular to ensure the phase coherence of multi-pass acquisition: the method initially evaluated for high frequency radar is to be validated for JuRa and Juventas when coupled with error model of orbit restitution.

### Advanced Radar Tomography

Tomography methods have also been developed for structural imaging. Although they appear totally different from SAR processing methods, it should not be forgotten that they are dealing with the same propagation physics, which are just described using a different formalism. These algorithms aim at reconstructing structural maps of the scene (positions and shapes of structures, without quantitative permittivity values) and rely on well-established inverse problem theory (Devaney 2012). In order to exploit both the amplitude and phase of the scattered field (in the time or frequency domain), diffraction tomography methods based on the scattered field observation equation (see Sect. 2.2) have been developed, taking full advantage of the spatial bandwidth of the measured field. These methods focus on the Green’s function (Gbur and Wolf 2001) and exploit the filling of the Ewald sphere (see Sect. 2.2).

**Fig. 6:**
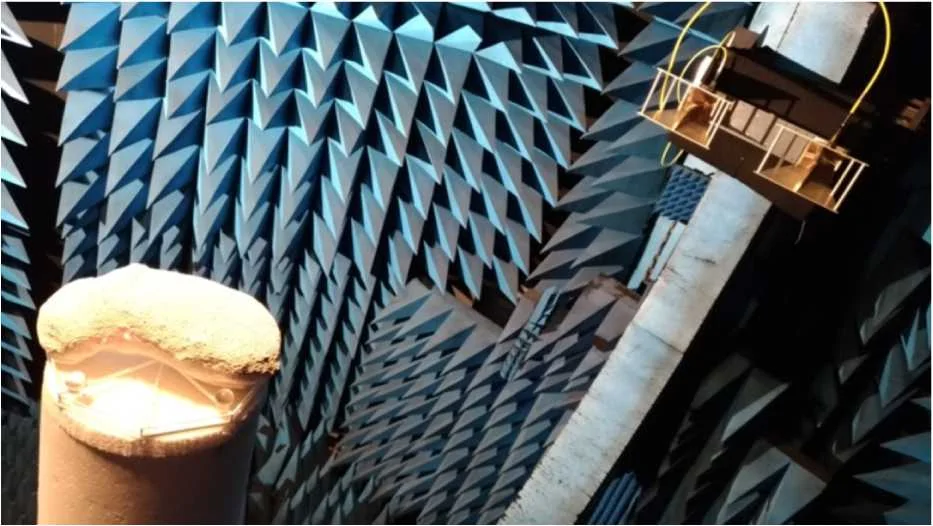
Measurement configuration in the anechoic chamber in Centre Commun Ressources Micro Ondes (Marseille, France) and asteroid ($25{,}143$) Itokawa analogue

**Fig. 7:**
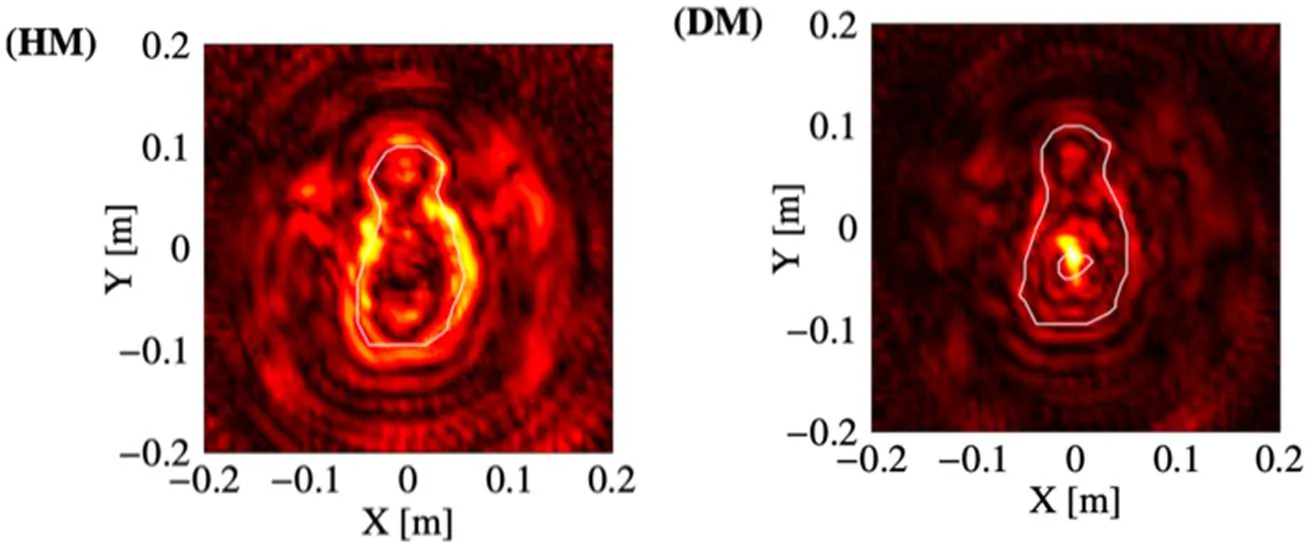
Slices of the amplitude of the reconstructed induced currents with Pseudo-inverse method in the frequency domain for both analogue with void (right) and homogeneous analogue (left) (from Dufaure et al. 2023)

In the context of the asteroid mission proposal, methods adapted to the specificity of the radar tomography in terms of permittivity contrast, size of the observed domain and geometry of the sensors were developed. Given the large size of the imaging domain and the resulting computational load, it is usual to seek a linear formulation of the Green’s function. As it turns out, the Green’s function at a given frequency establishes a linear relationship between scattered fields and induced currents . Accordingly, vectorial induced current tomography methods were designed with the objective to reconstruct maps of induced currents  rather than permittivity contrasts . Depending on the sampling on the Ewald sphere, the 3D map of the volumetric induced current can be obtained either using the 3D inverse Fourier transform of the scattered field measurement in the frequency domain (Eyraud et al. 2013) or using the Pseudo-Inverse operator and the adjoint of the Green’s function (Dufaure et al. 2023). These computationally light methods have successfully been applied to reconstruct the interior of asteroid analogues using a monostatic configuration as shown in (Fig. 6, Fig. 7).

In well-known classical first-order diffraction tomography algorithms, linearization of the Green’s function is performed with regard to the permittivity contrast . Since the Green’s function is a function of the unknown permittivity contrast, linearization is achieved through the Born or Rytov approximation (Zernov and Lundborg 1993). The scattered field vector is therefore assumed to be collinear with a known reference field vector associated to a chosen background permittivity contrast model. Using the incident field vector associated to the free space model is a straightforward and computationally light method which corresponds to the SAR processing presented above. Unfortunately, it is poorly adapted when imaging high permittivity contrast interiors mainly due to surface refraction and scattering. Accordingly, background permittivity contrast models closer to the true asteroid permittivity contrast are often preferred. For instance, asteroid shape models can be used to build background homogeneous asteroid permittivity contrast models. Associated reference vector fields to homogeneous asteroid models can account for surface scattering and refraction whereby improving the positions and shapes of scatterers within the asteroid. While it is possible to accurately compute Green’s functions of 3D homogeneous asteroid models (Berquin et al. 2025) this remains an intensive computational task. Numerical approximations using geometrical optics have been used to develop time domain imaging algorithms for asteroids (Hu et al. 2024; Shi et al. 2024). In Haynes et al. (2024) a numerical study with Pseudo-Inverse method in the frequency domain under the Born approximation and using the T-matrix formalism (Mishchenko et al. 1996) has been applied to reconstruct a 3D model of asteroid 99,942 Apophis in a bistatic configuration. A time-domain backpropagation algorithm combined with techniques for filtering the multiple diffraction and multiple paths contained in the input field (such as Truncated Singular Value Decomposition, pathlength or peak detection) has been applied to reconstruct a 2D numerical asteroid analogue (Sorsa et al. 2023; Yusuf et al. 2022).

In order to obtain quantitative values of the scene permittivity in complex cases, iterative non-linear inversion methods are required. Non-linear algorithms necessitate to compute Green’s functions associated to updated permittivity contrast  at each iteration and therefore remain prohibitively computationally expensive given the size of 3D asteroid domains. Non-linear iterative methods have been developed in the inverse problem community and investigated in recent years to image 3D targets (Eyraud et al. 2019). Numerical studies have been carried out to image the permittivity map of a 2D scale model of an asteroid, imposing the external shape and including prior knowledge on the background permittivity inside the model: with the Distorted Born iterative method (Haynes et al. 2021) and using a metric derived from optimal transport theory (Deng et al. 2022).

## JuRa Science Case

What do we know about the internal structure of these binary systems? Mainly information derived from the mechanical and thermal models of formation, evolution and stability of these bodies and their confrontation to surface properties from optical remote sensing. For Dimorphos, the physical modeling of the moonlet response to DART impact to reproduce optical observation was incredibly productive. Without wishing to make an exhaustive review of Didymos and Dimorphos after DART (see Michel et al. 2025, this collection for a detailed bibliography), we can sketch a JuRa-oriented overview and derive key questions to which JuRa ambitions contribute substantially.

### Didymos

Starting from the basics, Didymos is likely a rubble pile, an aggregate of debris resulting from the catastrophic destruction of the Baptistina family’s parent body 140 to 320 Myr ago (Masiero et al. 2012; Richardson et al. 2022). It would not be the direct descendant of this disrupted parent body, but rather the N^th^ generation resulting from collisional disruptions. As any member of the kilometric size asteroid family, Didymos is constantly being reconfigured under the effect of YORP accelerations (Fang and Margot 2012; Jacobson and Scheeres 2011). It is presently a binary system whose shape suggests that its moon was formed by the mass shedding from the main body and reaccumulating in orbit rather than by catastrophic fission (Barnouin et al. 2024).

**Fig. 8:**
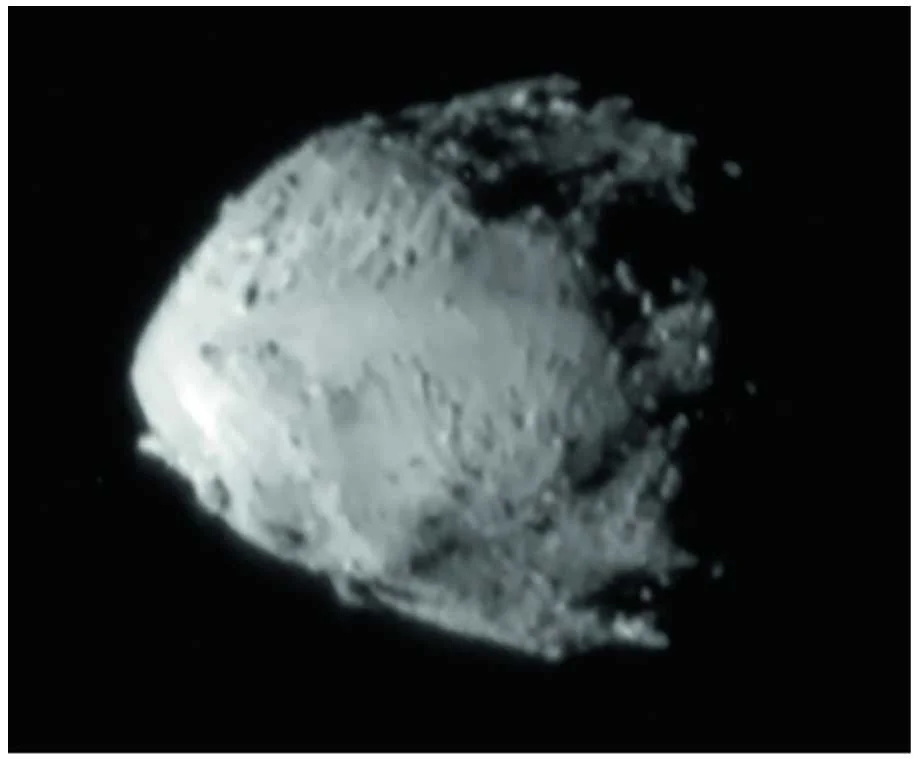
Didymos imaged by DRACO/DART before impact. Credit NASA/Johns Hopkins APL

Didymos (Fig. 8) presents a flat top-shape with geophysical units structured by latitude: an equatorial ridge characterized by low roughness without boulders larger than 5 m; in contrast to the polar regions undulated and rough with many large boulders in the range 10 to 160 m. At middle latitude, the transition zone is mixing both structures (Barnouin et al. 2024). This bulge corresponds to a surface geophysical structure produced by the regolith movements and the gradual mass shedding leading to the formation of Dimorphos (Vincent et al. 2024). The mean density is estimated to be 2800 ± 280 kg/m^3^, which leads to a 21 ± 8.5% porosity, assuming a grain density of 3550 kg/m^3^ corresponding to a L or LL chondrite. Such porosity is low, but may be significantly underestimated as the volume appears to be underestimated (Barnouin et al. 2024). With a fast spin period at 2.26 h, Dimorphos is close to its disruption spin barrier, while the question of the stability of the equatorial regions remains open due mainly to uncertainties in density (Campo Bagatin et al. 2024). Its dynamic state therefore does not place strong constraints on surface cohesive strength. The flattened shape of Didymos in response to YORP suggests a cohesive interior (∼10 Pa) covered by a weakly cohesive regolith (<1 Pa) that responds more strongly leading to progressive Dimorphos formation by mass shedding. This corresponds to a surface loss of 3-5 m (Barnouin et al. 2024). The 12.5Myr age from surface features is difficult to interpret, given that the surface is constantly being resurfaced by impacts and, to a lesser extent, by YORP spin-up (Campo Bagatin et al. 2024).

After DART, the rubble pile structure of Didymos seems unquestionable. However, the DRACO camera onboard DART was only able to observe the surface and provide an estimate of the size distribution of surface boulders down to the resolution limit, which does not give access to fine particles. It is thus essential to determine whether the surface distribution reflects that of the subsurface and of the interior or whether the subsurface and interior have different properties. In particular, the shallow subsurface with a latitudinal structure is the main area mobilized in response to impacts and YORP acceleration. This is the key area for understanding Dimorphos formation and the evolution of the binary pair, in particular the resurfacing and finally the context of any observation of the surface. It is therefore crucial to characterize these different terrains units in terms of block size, porosity, heterogeneity, if possible, their stratigraphy and finally to reconnect them with each other. It is also necessary to understand the connection between these surface formations and a more cohesive deep interior.

### Dimorphos

**Fig. 9:**
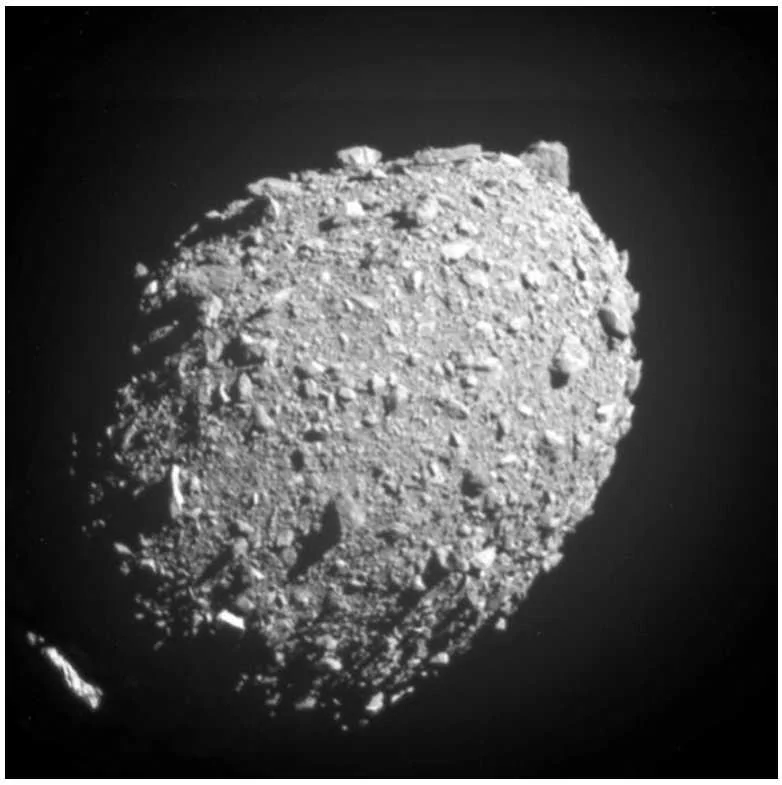
Dimorphos before the DART impact imaged by DRACO/DART. Credit NASA/Johns Hopkins APL

Regarding Dimorphos (Fig. 9), we must emphasize that Hera will reveal in the fall 2026 a very different Dimorphos than the one observed by DART before impact in 2022. This is one of the most exciting goals of the Hera mission. As a result of DART impact, Dimorphos is expected to have experienced a global reshaping with 1% or less of mass ejected but up to 8% displaced at the surface including the ejecta fallouts (Raducan et al. 2024). In addition to the orbital period change, the small moon should be in a tumbling state. Since relaxation times of such an excited state are poorly constrained, the rotation state in 2027 is therefore unknown, particularly regarding the 1:1 resonance (Richardson et al. 2024).

Speaking about the pre-impact Dimorphos, this is a more porous body than Didymos, with a macro-porosity of ∼35% and an unknown but estimated average density of 2400 kg/m^3^. The fraction of large boulders (2.5 m < size < 16 m) is lower than 40% in the impacted part of the moon and the surface does not show any fine debris (< 10 cm) (Barnouin et al. 2024; Raducan et al. 2024). The surface is globally uniform, with no obvious terrain units. It appears to be an accumulation of blocks with limited subsequent production of regolith by thermal fatigue and shock (Barnouin et al. 2024; Lucchetti et al. 2024; Robin et al. 2024). The surface age, estimated to be less than 0.3 Myr, is also ambiguous to interpret, since it may correspond to the formation of the moon, to resurfacing linked to an impact, or to episodic mass exchange with Didymos (Barnouin et al. 2024).

The characterization of the rubble pile structure of Dimorphos is fundamental to interpret the response of the asteroid to the DART impact, which will also be measured by Hera. This characterization also includes the possible presence and size of fine material. Each question has to be seen in conjunction with Didymos in order to identify any possible segregation in the process of Dimorphos’ formation or evolution. The aim here is to understand whether the homogeneity observed at the surface before DART impact is also a characteristic of its interior as well as the apparent absence of fine particles. We will thus be able to determine whether there is indeed no stratigraphy linked to regolithization or whether we observe features related to impacts: ejecta redeposits or internal compaction areas.

### JuRa Objectives

The ambition of JuRa is to probe the internal structure of the two asteroids, characterizing the degree of heterogeneity of the first hundred meters or more (Michel et al. 2022).

The first objective of JuRa is to characterize the interior of Dimorphos, to reveal its aggregate structure and to characterize its level of heterogeneity at scales from 20 cm to 20 meters (typical of the expected size of the constituent blocks and of the voids, the presence of fine dust or gravel filling the voids which constrain macro- versus micro-porosity). The spatial variation of the received signal should allow us to identify internal geological structures such as layers, larger blocks or voids, sub-aggregates, and significant spatial variation of the density, porosity or block size. Such information will provide constraints on the mechanical model of the DART impact process. However, it should be noted that a direct detection of the DART-induced internal mass redistribution in the near vicinity of the crater is not expected, considering the 10–15 meter JuRa expected 3D resolution.

The second objective is to estimate the average permittivity and to monitor its spatial variation in order to retrieve information on the moonlet composition and porosity. Radar bypasses the near surface alteration by space-weathering and thermal-cycling addressed by optical remote sensing. A full penetration of the whole moonlet in case of limited level of scattering would allow a direct measurement of the average permittivity and a 3D projection tomography of the permittivity (cf. Sect. 2.1), while a partial penetration will only provide an indirect estimation of the permittivity contrast in a diffraction tomography approach.

In the Didymos section, we saw it is crucial to do the same characterization of Didymos, to detect differences in structure and composition. A comparison of signal textures received from the main and the moon will demonstrate the presence of segregation processes during moon formation in a binary system, helping to discriminate between progressive versus catastrophic processes and to model the stability conditions of the system.

Finally, the radar data processing requires an accurate determination of the geometry of observation of the spacecraft and the observed target (orbits, rotations, libration, mutation). The geometry can then be constrained from radar data itself using autofocus methods. The possible contribution of JuRa to support the determination of the dynamical properties of the Didymos system will be evaluated.

JuRa being the first monostatic radar tomography of an asteroid, it is important to note the limited maturity of the technique. In other words, the internal structure of a binary asteroid and its radar signature remain largely unknown. This limits our capability to specify the instrument and to design a processing pipeline. This is also the ambition of JuRa.

If we now consider the landing phase, during which JuRa could operate depending on the resources available, the objective is to ensure an absolute measurement of the average permittivity based on the round-trip propagation time through the moon. Indeed, this configuration is much more favorable in terms of signal-to-noise ratio, antenna coupling and surface scattering.

## The Radar Definition

### Dielectric Model

The concept and the specification of JuRa are based on our knowledge of the dielectric properties of the two bodies. The dielectric permittivity of Didymos and Dimorphos depends on the mineralogy of their constitutive material. The binary system is an S-type asteroid (de León et al. 2010; Michel et al. 2016; Pravec et al. 2012) which corresponds to ordinary-chondrite meteorites. The expected dielectric properties are summarized in Table 2 as discussed in (Herique et al. 2018). For a review of the dielectric properties of meteorites and minerals in the context of radar sounding of small bodies, one can also refer to (Herique et al. 2016). It is important to understand that propagation losses in Table 2 only correspond to dielectric losses and do not take into account scattering losses: the decrease of the transmitted power wave due to scattering. This last effect would be the dominating one and remains difficult to estimate a priori but is one of JuRa’s goals.

**Table 2:** Dielectric properties from Herique et al. (2018). Dielectric losses $\alpha =0.091 \sqrt{\varepsilon} f\tan\delta \: d$ in dB where f is the frequency in MHz corresponds to the 1-way attenuation for a wave reaching a feature at distance d bellow the asteroid surface. This attenuation does not take into account scattering losses nor geometrical expansion of waves

	Bulk material	Average
Macro Porosity	0%	30%
$\varepsilon^{\prime }$	7 to 10	6 to 4.5
tan $\delta = \varepsilon ''$/$\varepsilon^{\prime }$	10^−2^	8 10^−3^
dielectric losses @ 60 MHz (one way)	16 dB/100 m	10 dB/100 m

To conclude, the question of the variability of the permittivity inside a given block at wavelength scale is clearly pending: meteorites laboratory measurements exhibit a large dispersion of measured permittivity (Herique et al. 2016) while they are mainly performed at a few centimeter scale using coaxial probe. Metric scale statistics have never been addressed properly, mainly due to samples size.

### Instrument Definition

JuRa is a monostatic Synthetic Aperture Radar dedicated to probe the internal structure of the binary asteroid. The main trade-off leading the instrument definition is the selection of the carrier frequency and the RF bandwidth: the lower the frequency, the better the penetration in terms of both dielectric losses and surface scattering losses while the volume scattering is poorly constrained as explained in Sect. 2.1. On the other hand, the higher the frequency, the wider the possible RF bandwidth and the better the resolution, the smaller the antenna size and the easier the antenna accommodation especially on a small satellite. For JuRa the carrier frequency is 60 MHz with nominal bandwidth (BW) of 20 MHz and an extended BW of 30 MHz without any signal-quality warranties (Table 3). This carrier corresponds to a wavelength of 5 m meaning dipole antenna consists in two booms of ∼1.25 m each which is compliant with a CubeSat accommodation. The nominal bandwidth corresponds to a third of the carrier which is challenging especially for the antenna matching, to provide an RF resolution better than 10 m in vacuum. Backscattering coefficient for rocky asteroids at 60 MHz strongly depends on the surface roughness and is thus difficult to extrapolate from Earth remote sensing. Rocky surfaces as in (Ulaby and Dobson 1989) suggest values down to -20 dB.m^2^/m^2^ at 45° for direct linear polarization and -30 dB.m^2^/m^2^ for cross polar. Assuming -16 dB losses for one-way propagation throughout the 160 m-large S-type and “low-scattering Dimorphos”, a sensitivity defined by a Noise Equivalent Sigma Zero (NES0) better than -60 dB.m^2^/m^2^ at the surface would allow us to probe the whole moonlet.

**Table 3:** JuRa main Instrument characteristics

Carrier Frequency	60 MHz	
Signal Bandwidth	20 MHz	30 MHz (extended)
Polarization	Full linear	
NES0	-60 dB.m^2^/m^2^	
Ranging resolution	10 m	
Electronics	95 × 95 × 94.8 mm^3^	1 kg
Antenna (4 booms)	50 × 44 × 32 mm^3^ folded, 1259 mm deployed	65 g each
Primary power	29 W average in operation	

**Fig. 10:**
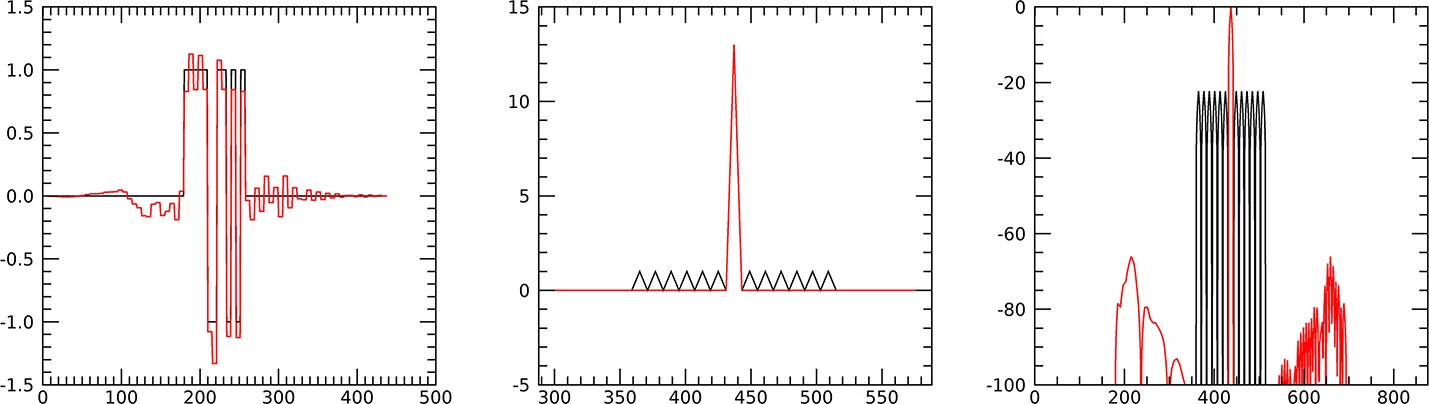
Optimal Filter (red) and matched filter (black) for a 13-symbol Barker code: Filter impulse response (left), compressed signal in linear (middle) and dB (right) scales

The radar will operate at a closest range of 2700 m to 750 m to Didymos and Dimorphos, respectively, during the main operational sequences. The commissioning and calibration during COMP phases and possibly INSP phases (Table 1) as well as the risk mitigation in case of navigation contingency require to provide capability to operate at largest distance up to 10 or 30 km. On the other hand, the optional operation during landing requires to operate a lower distance: the aim is to provide some capability to operate after landing as a Ground Penetration Radar (GPR) with the smallest blind zone. This need of versatility in terms of radar chronogram and close operation is the second driver of a radar definition for such a small body mission. This led us to consider a coded radar instead of a more classical chirp radar (Levanon and Mozeson 2004): the emitted signal is a binary code (−1/+1) constituted of a suite of symbols of 5 ns (20 MHz BW) modulated by the carrier (3 periods per symbol) and the received signal is match-filtered after demodulation to compress this code. The selection of the binary sequence and its length provide versatility in terms of minimum operation distance and also in terms of SNR even allowing to operate in pulsed radar with only one symbol transmitted in “GPR” mode. Such Binary Phase Shift Key codes (BPSK) are known for their relatively high side lobes limiting the Pulse-to-Side Lobe Ratio (PSLR) and instantaneous dynamics especially for short codes. This can be overcome using Barker code compressed by optimal filters proposed by (Griep et al. 1995). Figure 10 shows the optimal performances for a 13-symbols Barker code with a final PSLR better than 60 dB at the cost of a very limited SNR degradation (0.21 dB). These performances are more than sufficient, and the final performances will be limited by the instrument calibration. When applied to longer codes such as nested Barker codes, the optimal filter ensures comparable performances with the rejection of the side lobes far enough from the main pulse.

JuRa antenna is close to a half-wavelength cross-dipole. Its directivity is very low and the whole binary system is illuminated by the antenna: the Doppler bandwidth and centroids are independent, at the first order, of the antenna pattern of the instrument pointing while they are determined by the size and the rotation speed of the two observed bodies as shown Fig. 5. Size and rotation speeds are the parameters that determine the Pulse Repetition frequency (PRF) and on-board pre-summing, which completely changes the logic of the instrument design by regards to classical spaceborne SAR. At the shortest distances, the antenna pattern begins to affect the power received from the asteroids and would be considered for radiometry. The other impact of the antenna aperture is the link budget: a large fraction of the emitted power does not reach the target. This is acceptable due to the low relative speed and low operation distance.

Polarization is another key factor in antenna specification. Here again, the geometry of small bodies is a specificity: as the entire body surface is illuminated, the local vertical changes from one point to another in the field of view: it is not possible to choose a linear polarization that would be constant over the entire observed scene and locally constant throughout an observation sequence, which is essential for coherent SAR processing. To overcome this, the first idea was to use full circular polarization, transmitting one circular polarization and receiving both similar to ground-based radars such as Arecibo or Goldstone. In practice, we moved to a full linear polarization with two independent dipole antennas, two transmit channels and two receive channels. This solution presents a number of advantages: the first is technical, with a simplified matching network design, fewer insertion losses and better isolation of the two channels for a better cross-polar ratio; the second is risk mitigation, with partial Tx redundancy and the possible operation in degraded mode in the event of failure of one amplifier. Finally, the possibility of processing the two linear polarizations separately and compensating the gain of each dipole with a full 3D SAR processing ensures radiometry accuracy and the cross polar ratio during processing.

### Link Budget and Radiometry

At JuRa frequencies, the main noise source is the galactic synchrotron radiation, for which we took 20000 K at 60 MHz (Kraus 1986) which is conservative compared to some other models (De Oliveira-Costa et al. 2008). Then, for a given scenario, the signal to noise ratio on the surface for a resolution cell is $\textit{SNR}=M \frac{P_{r}}{\gamma _{0} B_{r}}$ with M the processing gain (number of accumulated measurements), $\gamma _{0}$ the galactic noise spectral density, $B_{r}$ the receiver bandwidth for each measurement and $P_{r}$ the power received from one resolution cell. $\textit{SNR}= \frac{M P_{t} G^{2} \lambda ^{2}}{\gamma _{0} B (4\pi )^{3} R^{4}} \,\sigma _{0} \, S_{r}$ with $P_{t}$ the transmitted power, G the antenna gain, R the distance to the surface and $\sigma _{0} \,S_{r}$ is the radar cross section of the resolution cell of a surface S. The Noise Equivalent Sigma Zero (NES0) is the limit of sensitivity equal to the sigma zero for SNR = 0 dB.

The spatial resolutions have to be approximated from simulation of the considered observation scenario in order to take into account the full-geometry and the large relative-bandwidth both in Doppler and in RF. The classic formula $S_{r} = \frac{c}{2 B_{r} \sin (i)}\ \frac{\lambda R}{2Tv}$ with c the speed of the light, $B_{r}$ the RF-bandwidth, i the local incidence, T the duration of the cell observation and v the orbital relative speed provides just an order of magnitude.

A 45-minutes long sequence of observation on a 2 km radius orbit then leads to a resolution ∼50 m at 45° incidence (worst case). This scenario provides a -60 dB.m^2^/m^2^ NES0 worst case for both the main and the moon assuming a 13-symbol long BPSK, a 0.8 Hz PRF and 1000 internal accumulation. Operation at larger distances is less favorable, which can be compensated for by a longer transmitted code.

## JuRa Instrument

### Instrument Concept

**Fig. 11:**
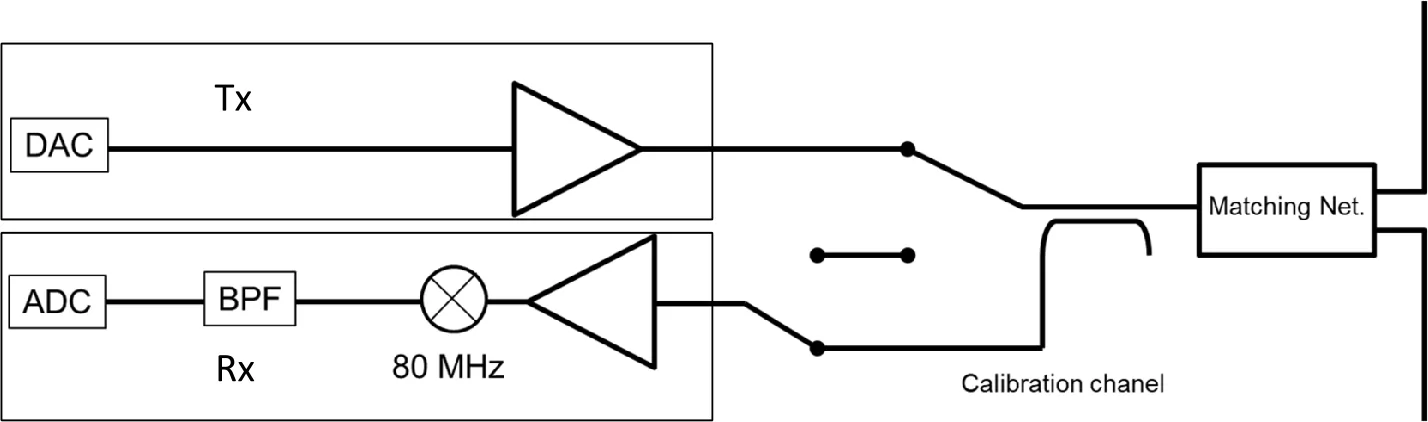
Schematic representation of the JuRa system design

JuRa is a monostatic coded radar (Table 3). The instrument concept (Fig. 11) is based on the direct generation of the code and its carrier by a 120 MHz chopper. This signal is amplified by the power amplifier acting as a filter to limit the transmission of harmonics. The received signal is mixed by an 80 MHz carrier and filtered before sampling at 120 MHz, after which the signal is digitally processed. During the transmission phase, the receiver (Rx) is connected to the transmitter (Tx) by a directional coupler, two attenuators and a switch (56 dB total losses) to provide a signal as calibration reference (Fig. 11). Two power meters are implemented before the matching network to provide a measurement of the forward and reflected power during the Tx phase to improve radiometric accuracy. During the Rx phase, the Rx channel is directly connected to matching network and antenna nearly without extra coupling losses.

JuRa is designed as a fully polarimetric radar. The two linear polarizations are totally separated with dedicated Tx channels, Rx channels and matching network connected to the two perpendicular dipole antennas.

**Fig. 12:**
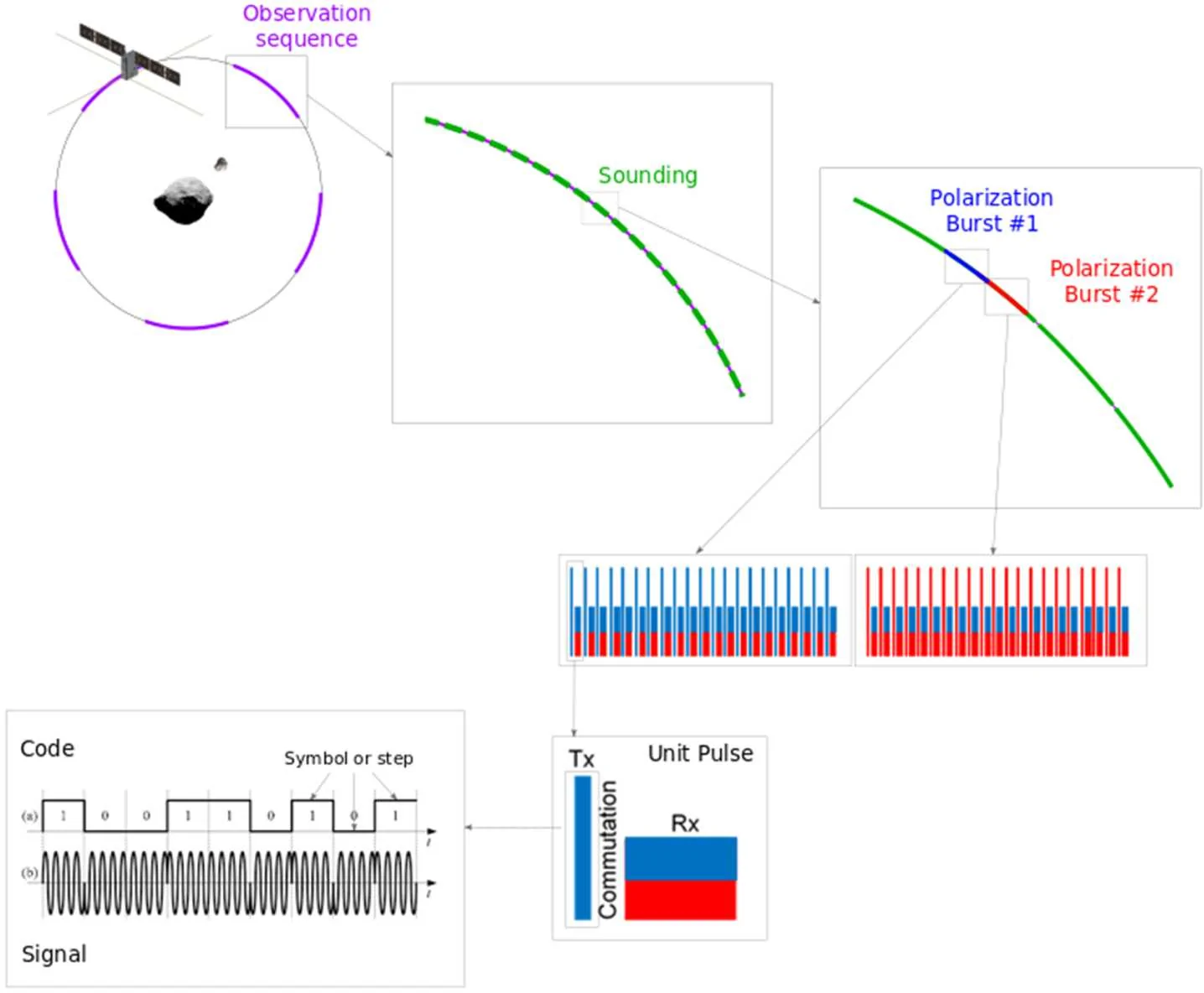
General concept of JuRa measurements

The measurement is an alternation of transmissions of one polarization by one Tx channel with the reception on both Rx channels corresponding to the co- and cross polarizations channels, then the second polarization is transmitted and received on both channels (Fig. 12). For each measurement point, the transmission and coherent accumulation of a sequence (burst) of unit pulses for each polarization increases the SNR, which remains negative before pulse compression. This low-pass Doppler filtering is limited by the highest Doppler frequency, which may be larger than the Doppler bandwidth in the case of the moon.

Depending on the final orbitography the main instrument parameter ranges are quantified by the min/max-values listed in Table 4. The instrument parameters are depending on each other’s. Typical values are calculated for the nominal operation scenarios.

**Table 4:** Main instrument setting min max and typical values. Keep in mind that these parameters are not independent

	Min	Typical	Max
Symbol duration (ns)	33.3	50	100
Carrier period / symbol	2	3	6
Code length (symbol)	1	13-169	1024
Number accumulation	1	1000	20000
PRF (Hz)	0.1	0.8	8
Tx power (W)		4	4
Rx windows duration (μs)		1-20	100
Signal sampling frequency (MHz)	10 IQ	20 IQ	120
Number of bits / sample	2	4	16

### Instrument Development Philosophy

The radar system includes six electronic boards of which four form the base system (Generic Software-Defined Radio, GSDR) including power distribution, system supervision, FPGA, clocks and digital/analog converters. Two additional boards cover the radio frequency (RF) frontend functions, namely signal transmission (Tx) and reception (Rx) and the necessary commutation between both functions.

The JuRa instrument team is composed of seven partners. The overall design and production of the GSDR system and housing was under the responsibility of EmTroniX Sàrl (ETX) while the radar specific RF sub-systems were developed and produced by Technische Universität Dresden (TUD) for the Tx and the Institut de Planétologie et d’Astrophysique de Grenoble (IPAG / UGA) for the Rx board. The partners were supported by Hensoldt Space Consulting for the product and quality assurance. Software and firmware have been implemented by ETX and Filip Záplata (FZ) respectively. The mechanical system of the antenna, both booms and deployment mechanism, was produced by Astronica (AST), while the antenna electrical design, matching network and motor filters were done by TUD. IPAG was in charge of the instrument specifications and final verification of the performances.

The payload was developed between the start of phase B in July 2020 and the delivery of the instrument to the platform in July 2024. The Juventas CubeSat platform is following the development rules of the ‘*New Space*’ industry, which are significantly more permissive than traditional approaches. This is a first attempt of a deep space mission using this approach for an ESA mission, and it required a special risk assessment philosophy.

To ensure a high degree of robustness, the JuRa team had to adapt both the ECSS requirements of the Hera mission and the New Space regulations to find a suitable compromise. The requirements for thermal, mechanical, and electromagnetic compatibility (EMC) were adopted directly from Hera via the Juventas requirements. The compromise concerned the selection of EEE parts and their radiation hardening. To meet the tight schedule and relatively modest budget, a large part of the electronic components was provisioned from automotive industry. Wherever necessary, radiation hardened or ruggedized versions (e.g. the FPGA and memory chips) were chosen and as far as possible technologies robust to radiations were selected. Twenty-two commercial off-the-shelf (COTS) components for critical functions were selected to be tested individually regarding single event effects (SEE). As several components failed the SEE tests, it was necessary to redesign the affected functions using other components. In addition, the JuRa sequencer and processing core module has been implemented using triple modular redundancy (TMR) and parity protected registers for critical real-time control and command functions.

Another compromise was made at design level, choosing to keep a low level of redundancy. The radar instrument consists mainly of a linear chain of electronic components, each of which represents a single point of failure. JuRa benefits from partial redundancy as it has two RF channels, one for each linear polarization. The EEE parts were selected with great care, as described above. The only fully redundant subsystem is the memory for the main software and IP storage. Three standard memories can be selected from which the system boots. In the event of bit corruption, a fourth, radiation-tolerant memory acts as a secure fallback. Nevertheless, increased risks remain regarding this development strategy.

Engineering models (EM) of radar electronics were built for concept verification, TID and mechanical testing, and interface and signal quality verification. Finally, the AIT was based on a proto-flight model approach. The PFM underwent acceptance testing concerning vibration, EMC, thermal vacuum and was fully calibrated during the first half of 2023 for integration into Juventas in fall 2023. The JuRa team produced three additional models to serve as flight spare, platforms for ground testing and validation, and for field tests and calibration.

### JuRa Electronics

**Fig. 13:**
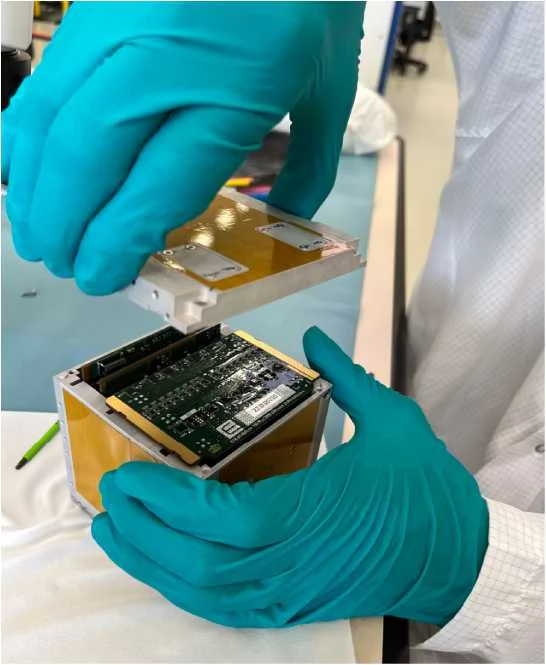
JuRa and flight model of the fully assembled JuRa electronics box.

**Fig. 14:**
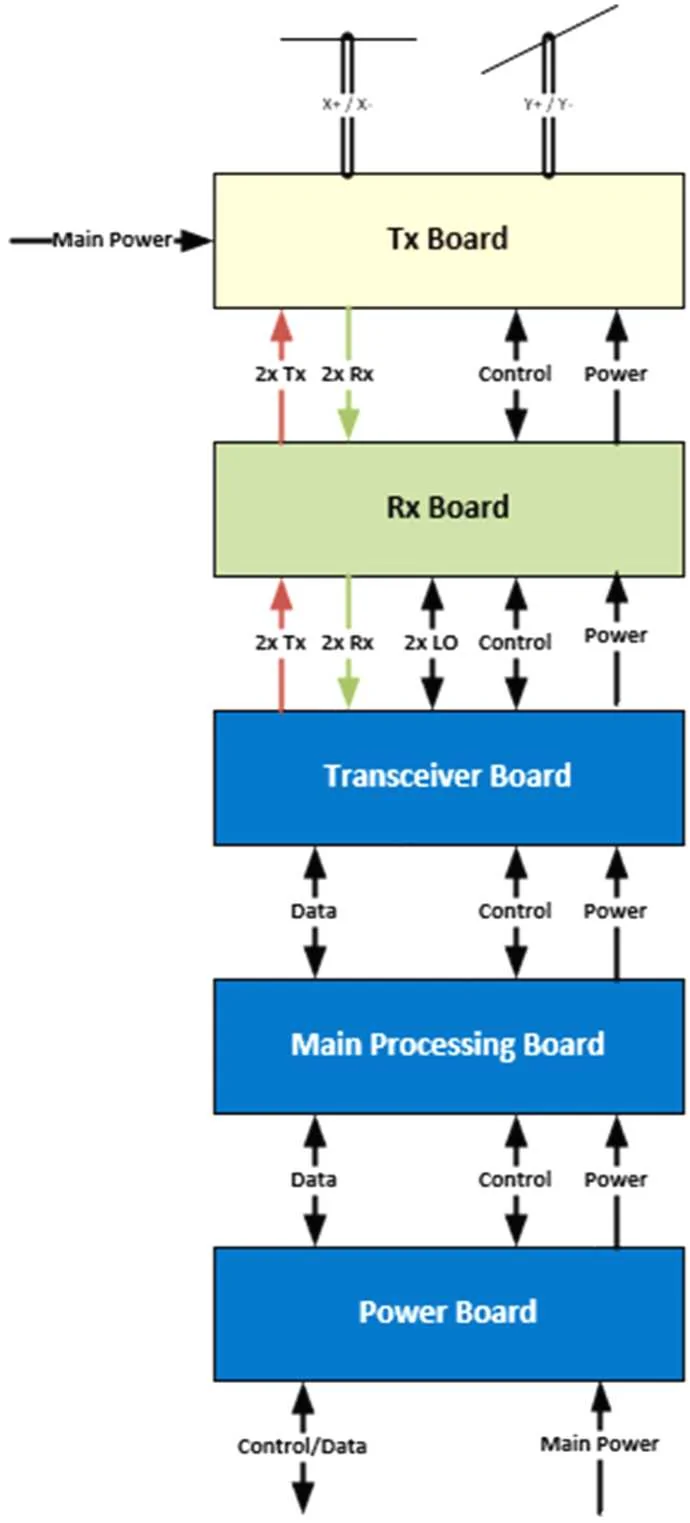
Arrangement of the PCBs within the JuRa electronics box

The JuRa electronics is integrated into a 1U mechanical housing (95 × 95 × 94.8 mm, Fig. 13, Table 3). The housing is divided into five brackets to which the individual printed circuit boards (PCBs) are attached, enabling good heat transfer from the PCBs to the housing. Components with high power dissipation have additional contact surfaces towards the housing including the top and bottom covers. The JuRa electronics is split into 6 PCBs (Fig. 14):

- The basis of the system is a software-defined radio (SDR) platform developed by EmTroniX, called Generic SDR (GSDR), which is divided into three main boards with specific functions. As the name suggests, this part of the design is generic in terms of hardware design.
- The power supply board (PB) serves as the main power supply for the SDR. It has an external power supply connection (28 V nominal voltage) as well as a user control and a data interface. This board also contains a dedicated controller that handles system monitoring (temperature, voltage, and current) as well as general functionalities such as those required for firmware updates.
- The main processing board (MPB) based on a Xilinx SoC (System-on-chip) implements all system components necessary for data processing and time-critical controls. The subsystem is designed to be radiation- hardened.
- The transceiver board (TRX) provides all necessary clocks for the system and contains a dual-channel analog to digital (ADC, 14 bits resolution, 120 MSPS) converter and a dual-channel digital to analog (DAC, 16 bits resolution, 120 MSPS) converter. The level of both ADC and DAC can be adjusted via digital step attenuators. In addition, the board provides two 80 MHz local oscillator signals (LO) for the receive chains.

All boards presented so far form the generic SDR platform. The actual application-specific part of the instrument is the radio frequency front end (RFFE), which consists of the receive board (Rx) and the transmit board (Tx). Both include two channels for reception and transmission, respectively.

The Rx board is implemented as a low-noise heterodyne converter (Fig. 11). The input signal of the board is first bandpass filtered (50–70 MHz) before being amplified by a low-noise variable amplifier stage consisting of an amplifier - step attenuator - amplifier chain. A subsequent low-pass filter acts as an image rejection filter before the signal is mixed to a center frequency of 20 MHz. The mixing stage is implemented with a high-side LO injection. The 80 MHz LO signal is provided by the TRX board to down-convert the input signal to a center frequency of 20 MHz (IF = LO – RF). A chain of low-pass filter, amplifier, and low-pass filter is then used to suppress the upper sideband of the mixer’s output signal and further amplify the down-converted signal.

The Tx board handles signal amplification, impedance matching, and acts as a signal and RF interface to the antennas. One of its main functions is therefore a switching matrix for switching between transmit and receive phases, and allow the calibration channel acquisition during transmission and power measurements (reverse and forward). In receiver mode, the antenna connection is switched to the input of the Rx board, which is fed from the Tx board. During transmission, the signal from the DAC of the TRX board is low-pass filtered, amplified to 36 dBm and matched to the antenna impedances.

### Onboard Software

The JuRa on-board software is composed of two parts: a real-time “radar unit” implemented on FPGA and an application processing unit (APU) implemented on the microprocessor.

The radar unit sequences the JuRa measurements and performs all real-time signal processing. The Tx signal consists of multiple repetitions of a pseudo-random (PN) sequence in a specific pattern. All of this is controlled internally by a sequencer, which also synchronously controls the Rx operations using a 120 MHz clock. The sequencer also includes the system for controlling the hardware modules such as RF switches or RF attenuators, which must rely on highly-synchronous and precise control timing.

**Fig. 15:**
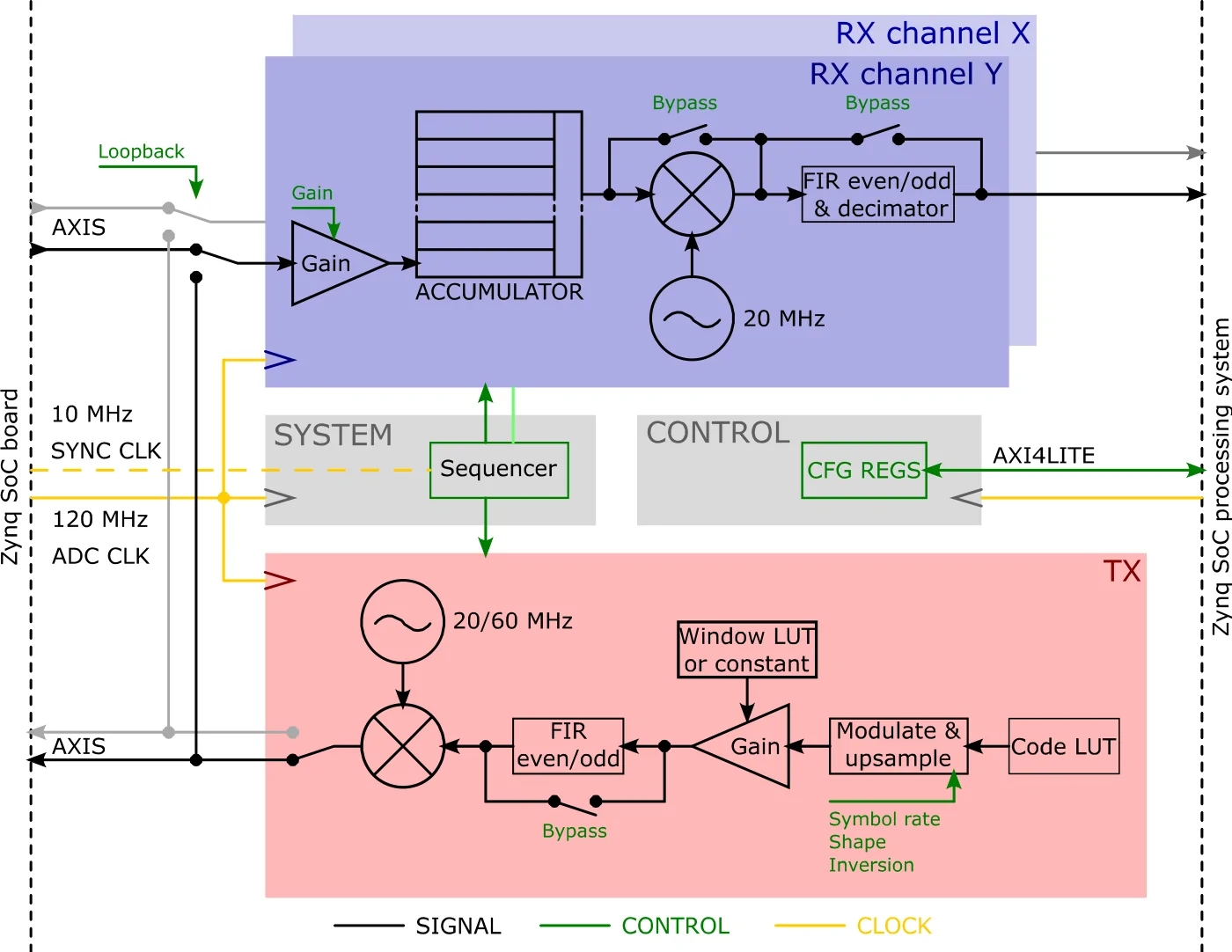
Block diagram of JuRa signal processing

Figure 15 shows a block diagram of the signal processing implemented in the radar unit. The radar transmits the X and Y polarization one after the other, in two separate bursts. This allows the implementation of a single Tx signal generator forwarded to the X or Y channel. The PN sequence can be arbitrarily configured by the user at any time before the sounding, giving a high degree of versatility during operations.

The radar uses BPSK modulation, thus the binary sequence is made of ±1 states. In addition, the signal is upsampled based on selected symbol rate, corresponding to the 10/20/30 MHz bandwidths. Signal amplitude can be shaped sample by sample or globally. An even or odd type digital filter removes the sidebands and smooths out the pulse. The harmonics generated by the square wave function of the DAC are largely filtered out by the power amplifier.

The receiver Rx samples are first accumulated inside a same burst (see Sect. 5.1), coherently added to improve the signal-to-noise ratio. Then, the downconverter module acts as a 20 MHz digital IQ mixer followed combined with a decimation filter in a single algorithm. Both the IQ demux and the decimation filter can be bypassed, to access the very raw signal at accumulator output, mainly for calibration purposes.

The accumulator and filter internally increase the dynamics of the digital signal: up to 48-bit output samples are supported. The output signal from real-time radar unit is truncated to 16 bits before being sent to the APU. On the APU, operating in-between two soundings, the circular polarization can be computed from all linear ones. Signals can then be optionally truncated again, down to 2 bits (typical nominal configuration is 4 bits) to eventually form the final science telemetry.

### JuRa Antenna Design

JuRa’s fully polarimetric antenna system consists of two crossed dipoles. Each dipole is composed of two single booms (e.g. monopoles) fed with two identical signals that are phase-shifted by 180 degrees (Table 3).

**Fig. 16:**
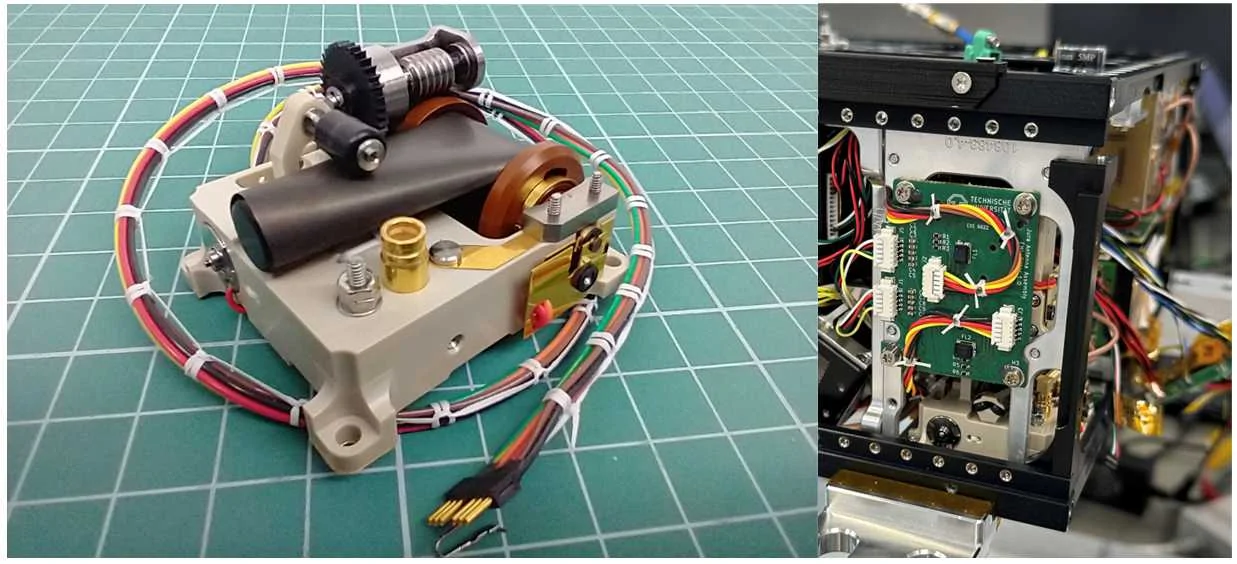
Flight Model of one of four JuRa antenna arms in stored configuration (left) and antenna and motor filter integrated inside the CubeSat (right)

Astronika produced a motor-driven deployment and retractable mechanism for monopoles, based on inherited designs. JuRa monopoles (Fig. 16) are deployed after the Juventas CubeSat separates from the Hera spacecraft. The ability to retract JuRa antennas was implemented to enable a proper landing on Dimorphos after Juventas successfully operates around Didymos and Dimorphos, allowing science operations with the GRASS gravimeter and, opportunistically, with JuRa. In retrospect, this retractability was useful for optimizing the input impedance of the antenna during the commissioning.

A single JuRa antenna unit (Fig. 16) consists of structural elements, a reel with antenna tape, a DC motor, a custom gearbox, a one-way clutch, and a limit switch system with shaft nut. The overall dimensions are 50 mm × 44 mm × 32 mm. The weight of a single antenna unit is 65 grams (Table 3). The antenna structure (housing) is manufactured out of plastic, while most of the metallic parts are grounded to reduce parasitical capacitances. To prevent coupling between the motor electronics and the JuRa RF radar signal path motor filters are designed and implemented on each antenna unit (Fig. 16).

The accommodation study allowed to optimize the cross-polarization ratio and to minimize the coupling between antennas and the CubeSat structure and solar panels. JuRa antennas are mounted in pairs in the corners of the S/C (Fig. 1).

**Fig. 17:**
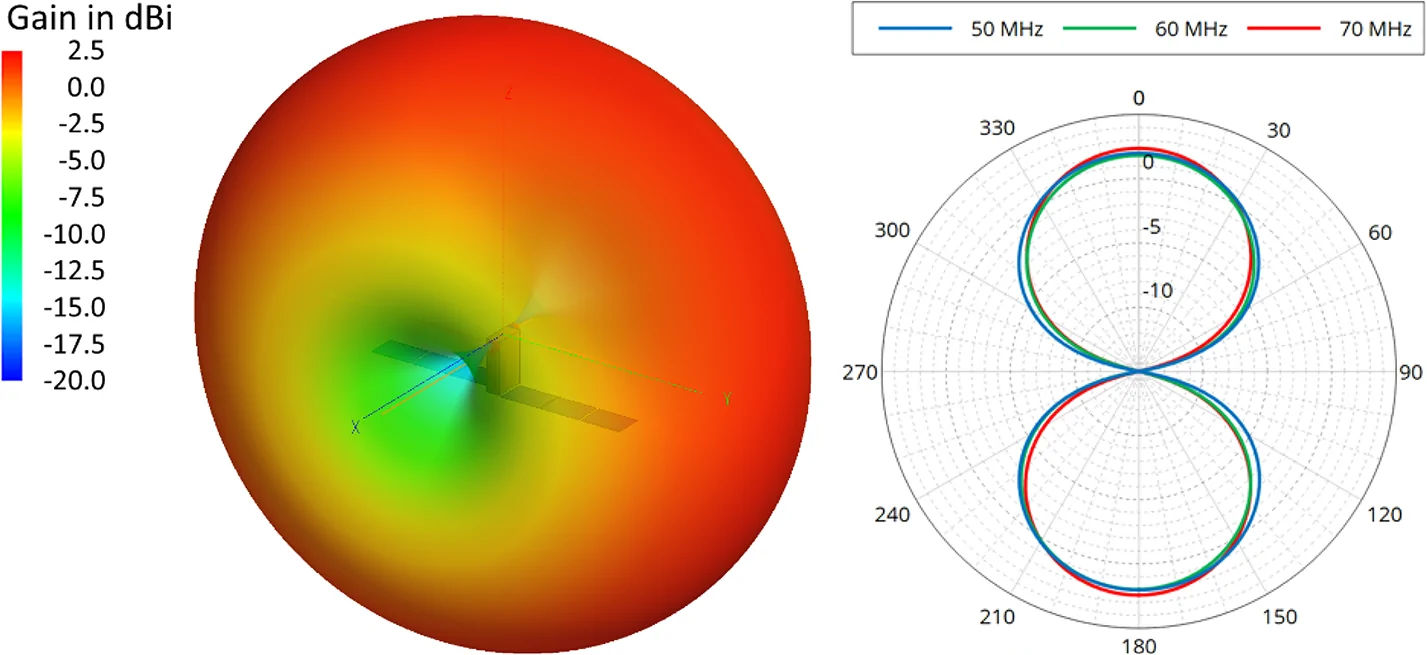
Typical simulation results of the JuRa antenna radiation pattern. Left: 3D radiation pattern of the dipole perpendicular to the solar panels at 60 MHz. Right: E-plane cut (0^∘^ equal to nadir) for different frequencies

Figure 17 shows simulation results of one of the 3D dipole antenna patterns at the center frequency of 60 MHz, the E-plane cut for different frequencies. The initial requirement of isolated solar panels from the spacecraft structure was finally not met which means that the solar panel has a significant impact on the antenna for Y polarization. This coupling breaks the antenna symmetry and leads to a following deployment strategy for the Y-axis dipole: during on-asteroid commissioning it will be partially retracted to optimize the matching and improve the resulting antenna pattern.

A matching network was developed to match the input impedance of the dipoles to 50 Ohms across the entire JuRa bandwidth. A critical point here is the measurement of the input impedance of JuRa antennas on the spacecraft at low frequencies between 40 and 80 MHz. The measurements on mockups were performed through drone tests and in an anechoic chamber, mainly to confirm the simulation techniques and simulation models used. Additional indoor tests of the JuRa flight spare electronics and antenna system were conducted to prepare in-orbit antenna deployment and optimization.

### Ground Test Equipment

**Fig. 18:**
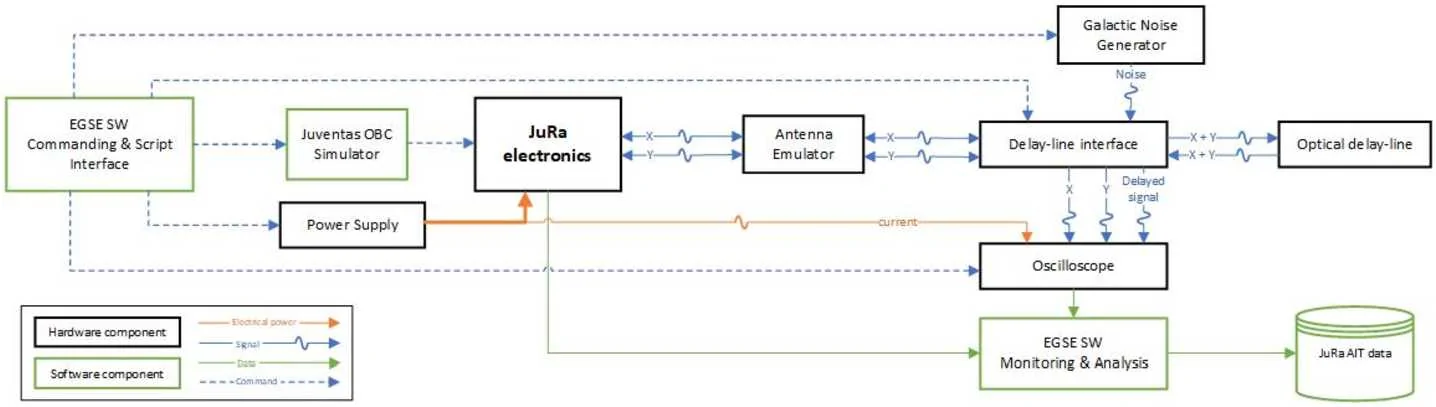
Block diagram of JuRa EGSE

The ground equipment for JuRa-AIT campaigns enables the radar experiment to be simulated in a laboratory or test environment. It consists of transportable hardware and software components for controlling them (Fig. 18). JuRa generates and transmits an RF signal via its +/-X and +/-Y antenna outputs. These outputs feed the antenna emulator, which taps a small portion of the signal power and sends it to the delay line interface. There, the X and Y signals are combined into a single line and split to the oscilloscope and the optical delay line. This module simulates the round-trip time of the radar wave to and from the asteroid and can be set to various simulated distances. Upon their return, the delayed signals are again split into X and Y channels and redirected to the antenna emulator and JuRa electronics. The delayed signals are also sent to a third channel of the oscilloscope. At this stage, it is possible to inject simulated galactic noise into the emulated reception signal. JuRa’s global power consumption is monitored on the fourth channel of the oscilloscope.

All these components are commanded, automated, and synchronized by the EGSE software suite based on the GAUSS framework developed by the CNRS. The test procedures are defined by a series of deterministic command scripts sent via the Juventas on-board computer simulator. The JuRa output data, consisting of digitized science data signals, calibration signals, housekeeping data, and acknowledgments, are captured by the monitoring and analysis tool for real-time visualization and stored in a database for archiving. This tool provides signal displays and high-level indicators of instrument status and performance quicklook.

### JuRa Data

The instrument is commanded by a mission table that defines an operation sequence with the Tx code loaded from an updatable library, the PRF, the Tx power level, the reception window, and the coherent accumulation, as well as the telemetry format which is versatile to allow for data volume optimization across the mission phases.

Science signals correspond to the received signal, demux around 20 MHz, sampled at 120 MHz, and then IQ demuxed. The sampling frequency of the IQ signal can be selected from 10, 15, 20, or 30 MHz. Accumulated signals before IQ demux can also be downloaded, mainly for ground calibration and instrument investigation purpose. The Rx window is defined by its t_0_ and duration. The number of bits can be set from 2 to 16 per sample using truncator with a framing factor. It was not possible to implement block adaptive compression within the constraints and timeline of the JuRa contract.

In terms of polarization, the whole acquisition is performed using full linear polarization (2 direct polar and 2 cross polar). To reduce the volume of data, it is possible to mimic circular polarization with one circular transmitted and two polars received. This on-board calculation halves the data volume at the cost of a loss of radiometric accuracy especially when moving away from the main axis of the crossed dipole. The solar-array coupling and antenna unbalance (Sect. 5.5) will further degrade this radiometric accuracy. In practice, this mode, intended for the first phase of operation, should be used sparingly.

In addition to the received signal, the internal calibration signal for each channel (Fig. 11) can be downloaded on demand. It is planned to perform a few internal calibrations per measurement sequence.

It is also possible to measure the forward and reverse power at the input of the matching network using two powermeters to ensure radiometric accuracy. This requires a specific transmission mode, which is longer than a normal pulse. This dedicated measurement is performed on request, at least for each measurement sequence.

## Conclusion

JuRa was launched in October 2024. In-cruise commissioning shows good instrument behavior and lower noise levels than in the laboratory. Obviously, the instrument’s emission section cannot be commissioned until Juventas has been released and the antennas deployed. Ground calibration data are currently being analyzed. This analysis is complicated by the fact that matching network differs for each antenna and is integrated into the electronic box. This analysis requires a correction of the antenna emulator. This post-processing is currently being validated. The final implementation of the calibration is scheduled for the second half of 2026 and will include antenna lobe modeling.

The cruise is also dedicated to the preparation of the operations. The objective is to cover as much of the observation geometry as possible, in other words to sample the Ewald sphere for Didymos and for Dimorphos as effectively as possible (Sect. 2.2). Of course, the limited data volume and orbital mechanics constraints mean that it is not possible to cover the whole spheres with Nyquist’s criteria. Both bodies will be undersampled: the undersampling strategy will be studied in 2026 based on the tomographic inversions presented above. In particular, for Didymos we can imagine focusing on specific areas of interest such as the equatorial ridge to image it with the best resolution while relaxing the resolution in some other areas. For Dimorphos, its size means that it will undoubtedly be handled globally.

The limitation of this optimization and the complexity of the operations come from the difference between the planned orbit and the flight one. Juventas station keeping is challenging due to the limited precision of the maneuvers performed by its propulsion system and Didymos’ low gravity with regard to perturbation strength. This results in an expected drift of a few days between the predicted orbit and the flight one. This is the situation for any spacecraft orbiting a small body, but it is undoubtedly even more critical for a CubeSat. In practice, this means that we are not flying in the expected geometry. This requires the implementation of a very short-term planning loop (VSTP) to tune the observations in terms of instrument settings and operation timing.

In general, SSTO is a very favorable orbit for radar operations since, with an almost constant orbital radius for a given operational phase, it should allow operations to be carried out with a stable instrument setting during this phase, making short-term planning simple. However, for Didymos, the binary system makes the problem more complex. The variation in the distance between Dimorphos and Juventas is significant over time, the Doppler bandwidth too (Fig. 5): an instrument setting that would cover all possible geometries within its operating margins would require an extended reception window associated with a high PRF. This would result in a more than twice increase in the volume of data per observation and the corresponding reduction in the number of observations which is not acceptable. It will therefore be necessary to update the instrument settings in the VSTP. The detailed implementation for acquisition optimization is under discussion at the operations centers and will be detailed in a follow-up paper.

Radar tomography is JuRa’s primary objective, and the instrument’s design was driven by this objective. With this design, the acquired data can be analyzed using an altimetric approach, allowing for a distance measurement between Juventas and both Didymos and Dimorphos with an accuracy better than 10 meters for a ground footprint of a few tens of meters, depending on the local curvature of the asteroid’s surface. This ranging measurement depends on the Juventas orbit, the dynamics of the two asteroids, and their surface shape. We are currently investigating how to use this measurement to support orbit determination, dynamic state modeling, and shape modeling especially for permanent-night areas. Similarly, multipass interferometry is currently under investigation. In both cases, their use remains prospective, and at a minimum, they will be used during radar pre-processing for measurement’s orbit refinement and for SAR-data co-registration. Another product from Jura is a surface map of the backscattering coefficient, which is directly related to surface roughness at a wavelength scale of 0.5 to 50 meters.

Last but not least, we must also mention RAMSES the mission to Apophis to be launched in April 2028 (Küppers et al. 2024). The Apophis RAdar (ARA) is currently being developed to instrument the Farinala cubesat (Herique et al. 2026). The instrument is almost a copy of JuRa, with a modification of the electrical interfaces and communication protocols to suit the different platform, a redesign of the antenna accommodation and the matching network. This second instrument will benefit greatly from JuRa’s heritage in terms of science operation planning and scientific analysis too.
